# Mitochondrial resilience: a convergent framework for pathogenesis and neuroprotection in Parkinson’s disease

**DOI:** 10.3389/fphar.2026.1886917

**Published:** 2026-08-24

**Authors:** Oscar Arias-Carrión, Magdalena Guerra-Crespo, Luis O. Soto-Rojas, Yeminia Valle, Emmanuel Ortega-Robles, Daniel Ortuño-Sahagún

**Affiliations:** 1 División de Neurociencias Clínica, Instituto Nacional de Rehabilitación Luis Guillermo Ibarra Ibarra, Mexico City, Mexico; 2 Tecnologico de Monterrey, Escuela de Medicina y Ciencias de la Salud, Mexico City, Mexico; 3 Laboratory of Regenerative Medicine, Department of Physiology, Faculty of Medicine, National Autonomous University of Mexico, Mexico City, Mexico; 4 Laboratorio de Investigación en Neurociencias y Enfermedades Neurodegenerativas (LINEN), Carrera de Médico Cirujano, Facultad de Estudios Superiores Iztacala, Universidad Nacional Autónoma de México, Tlalnepantla, Mexico; 5 Laboratorio de Neuroinmunobiología Molecular, Instituto de Neurociencias Traslacionales, CUCS, Universidad de Guadalajara, Guadalajara, Mexico

**Keywords:** alpha-synuclein, LRRK2, mitochondria dynamics, mitochondrial quality control, mitophagy, neurodegeneration, Parkinson’s disease

## Abstract

Parkinson’s disease (PD) is traditionally described as a dopaminergic neurodegenerative disorder driven by α-synuclein aggregation and selective neuronal loss in the substantia nigra pars compacta. While this characterization captures the core clinical and pathological features, it does not fully explain disease initiation and progression. Converging evidence from human genetics, cellular and structural biology, and systems neuroscience now supports a unified framework in which PD results from the progressive erosion of mitochondrial resilience. Here, mitochondrial resilience denotes the capacity of neuronal mitochondrial networks to withstand stress and recover bioenergetic and cellular homeostasis through coordinated quality control, metabolic adaptation, and organelle communication. Rare, high-impact monogenic mutations in *PINK1*, *PRKN* (encoding Parkin), PARK7 (*DJ-1)*, *LRRK2*, and *SNCA*, along with common risk variants identified in genome-wide association studies, converge on interconnected pathways that govern mitochondrial quality control, bioenergetics, organelle dynamics, and cellular stress responses. These vulnerabilities are most pronounced in the highly energetic dopaminergic neurons of the substantia nigra, where sustained calcium cycling, high bioenergetic demand, and environmental stressors increase cellular susceptibility. Research has moved beyond early observations of respiratory chain impairment and oxidative stress to reveal context-specific disruptions in PINK1/Parkin-mediated mitophagy, lysosomal trafficking, mitochondrial-derived vesicle dynamics, and neuroimmune signaling. This integrated framework reframes PD as a disorder of impaired cellular maintenance rather than solely a consequence of late-stage degenerative processes. It provides a translational shift from mechanism-based biomarkers to early detection of mitochondrial failure and supports therapeutic strategies aimed at restoring mitochondrial function and resilience, offering a direct route to disease-modifying neuroprotection in PD and potentially other neurodegenerative disorders.

## 1 Introduction

Parkinson’s disease (PD) is a clinically and biologically heterogeneous neurodegenerative disorder, classically defined by its cardinal motor features—bradykinesia, rigidity, resting tremor, and postural instability—collectively termed parkinsonism (Bloem et al., 2021). These motor symptoms primarily result from the progressive loss of dopaminergic neurons in the substantia nigra pars compacta, leading to dopamine depletion and widespread dysfunction within basal ganglia–thalamocortical circuits (McGregor and Nelson, 2019). However, PD is increasingly recognized as a multisystem disorder with clinical complexity extending beyond the dopaminergic system.

The pathological hallmark of PD is the accumulation of misfolded α-synuclein within neuronal Soma and processes, forming Lewy bodies and Lewy neurites (Zhang et al., 2026). Despite their historical prominence, the pathogenic significance of Lewy bodies remains debated. α-Synuclein deposition occurs in individuals without parkinsonism, while certain genetic or drug-induced parkinsonian syndromes show minimal or absent Lewy pathology (Adler and Beach, 2016; Beach et al., 2023; Arias-Carrion, 2026). The presence of early non-motor manifestations–autonomic, sensory, sleep, and neuropsychiatric symptoms that often precede motor onset by years–further illustrates that neurodegeneration in PD involves multiple neuronal populations and is not confined to the nigrostriatal pathway (Postuma and Berg, 2019; Pena-Zelayeta et al., 2025; Arias-Carrion, 2026). These non-motor features contribute substantially to disability and often prove refractory to dopaminergic replacement, exposing critical limitations of current symptomatic therapies.

Sporadic PD, which accounts for most cases, arises from the complex interplay of genetic susceptibility, aging, and environmental exposures. Advanced age remains the strongest demographic risk factor, and ongoing global population aging forecasts a marked increase in PD prevalence and societal burden in the coming decades (Dorsey et al., 2018; Arias-Carrion, 2026). Advances in human genetics have dismantled the historical distinction between familial and sporadic forms: rare monogenic mutations and common risk variants identified by genome-wide association studies converge on a restricted set of cellular pathways. Notably, genes such as SNCA and LRRK2 act as both high-penetrance Mendelian causes and modifiers of population risk, highlighting shared disease biology across clinical subtypes (Nalls et al., 2019; Blauwendraat et al., 2020; Arias-Carrion et al., 2026).

Convergent mitochondrial and lysosomal dysfunction has emerged as a shared mechanistic driver in both genetic and sporadic PD (Lucchesi et al., 2025). Findings from genetic models, patient-derived induced pluripotent stem cells, and multimodal omics have revealed recurring themes of mitochondrial dysfunction, impaired proteostasis, lysosomal–autophagic deficits, and defective cellular quality-control pathways as central drivers of neuronal vulnerability (Quinn et al., 2020; Lizama and Chu, 2021; Caldi Gomes et al., 2022; Chen et al., 2023). These processes interact in self-amplifying cascades that promote oxidative stress, bioenergetic collapse, and proteotoxic burden. Polygenic contributions from mitochondrial function-associated genes further reinforce mitochondrial pathways as key determinants of sporadic PD risk (Dehestani et al., 2022). Both monogenic and idiopathic PD converge on mitochondria as a central nexus of pathogenic stress, evidenced by deficits in PINK1–Parkin-mediated mitophagy, complex I impairment, and broader quality-control networks (Pickrell and Youle, 2015; Burbulla et al., 2021; Henrich et al., 2023).

Emerging data also identify intercellular mitochondrial transfer as a compensatory mechanism that can enhance neuronal resilience within glial–neuronal networks (Piao et al., 2026). Collectively, these advances necessitate a fundamental reframing of PD: rather than a late-stage dopaminergic motor disorder, it is best conceptualized as a progressive multisystem synucleinopathy initiated by convergent molecular vulnerabilities that precede clinical diagnosis by years or decades.

In this review, mitochondrial resilience is defined as the capacity of a neuronal mitochondrial network to withstand a defined stress, limit the resulting functional disruption, and restore bioenergetic and cellular homeostasis after the stress is removed. It is therefore a dynamic stress–response–recovery property rather than a synonym for preserved mitochondrial function under basal conditions. At the systems level, resilience depends on the coordinated activity of mitochondrial quality-control pathways, respiratory and substrate reserve, metabolic plasticity, redox and calcium regulation, inter-organelle signaling, and glial–neuronal support (Zong et al., 2024). This concept is related to, but distinct from, mitochondrial quality control and bioenergetic reserve. Mitochondrial quality control comprises the mechanisms that survey, repair, segregate, or remove damaged mitochondrial components, including proteostasis, fusion and fission, mitochondrial-derived vesicles, biogenesis, and mitophagy. Bioenergetic reserve, commonly estimated as spare respiratory capacity, describes the additional respiratory output available above basal demand at a particular time. These processes are determinants of resilience, whereas resilience refers to their integrated functional consequence: whether the neuron can preserve or recover ATP production, mitochondrial membrane potential, calcium handling, and cellular function following stress.

This review integrates current evidence positioning the progressive loss of mitochondrial resilience at the core of PD pathogenesis. It examines the mitochondrial defects that reduce stress tolerance and recovery in sporadic and genetic disease, dissects the molecular machinery involved, addresses persistent uncertainties, and explores how this framework may guide biomarker development and therapeutic strategies aimed at preventing irreversible mitochondrial and neuronal failure.

## 2 Mitochondrial dysfunction as a core hallmark of sporadic Parkinson’s disease

### 2.1 Complex I deficiency and impairment of the mitochondrial respiratory chain

The connection between mitochondrial dysfunction and parkinsonism was first identified in the 1980s, when accidental exposure to the neurotoxin 1-methyl-4-phenyl-1,2,3,6-tetrahydropyridine (MPTP) caused acute, irreversible parkinsonian syndrome in young adults. After crossing the blood–brain barrier, MPTP is converted to 1-methyl-4-phenylpyridinium (MPP^+^) and selectively taken up by dopaminergic neurons via the dopamine transporter, where it accumulates in mitochondria and strongly inhibits complex I of the electron transport chain (Langston et al., 1983). Although MPTP toxicity does not replicate the gradual progression of sporadic PD, its discovery established mitochondrial complex I inhibition as a plausible pathogenic mechanism and provided a durable experimental model still used to evaluate disease mechanisms and candidate therapies (Bove and Perier, 2012; Jackson-Lewis et al., 2012). The principal mechanisms of mitochondrial dysfunction implicated in sporadic PD are summarized in Table 1.

**TABLE 1:** Mechanisms of mitochondrial dysfunction in sporadic Parkinson’s disease.

Pathogenic process	Key molecular events	Biochemical consequences	Evidence and models
Complex I deficiency and respiratory impairment	MPTP → MPP^+^ uptake via DAT → mitochondrial accumulation → *complex I inhibition*	↓ NADH oxidation → ↓ proton pumping → ↓ ΔΨm → ↓ ATP synthesis; ↑ vulnerability to oxidative modification of complex I subunits; impaired OXPHOS assembly	MPTP primate/human cases; rotenone models; post-mortem SN and cortex: ↓ complex I activity + oxidized subunits; peripheral tissues: ↓ platelet complex I
Environmental mitochondrial toxins	Acetogenins (annonaceous fruits) → direct complex I inhibition	Chronic inhibition → bioenergetic stress in dopaminergic neurons	Epidemiological links to atypical parkinsonism in Caribbean populations
Early dopamine dysfunction due to mild respiratory deficits	Partial complex I loss (e.g., Ndufs4 deletion) → ↓ ATP → impaired VMAT2 loading → ↑ cytosolic dopamine metabolism	↑ Dopamine turnover; ↓ evoked dopamine release; ↑ oxidative metabolites	Conditional knockouts (dopaminergic-specific) demonstrating presynaptic defects preceding neuron loss
mtDNA instability and defective genomic maintenance	POLG proofreading loss → accumulation of point mutations and deletions → impaired mtDNA replication fidelity	Progressive, ETC., defects (esp. CI and CIV), premature ageing phenotypes; ↓ OXPHOS capacity in neurons	POLG mutator mice; POLG1 variant carriers with parkinsonism
TFAM loss/mtDNA depletion	TFAM deletion in DA neurons → ↓ mtDNA copy number → ↓ transcription of mitochondrial genes → ETC., collapse	Motor impairment; nigral degeneration; L-DOPA responsiveness mimicking PD	Conditional TFAM knockout models
Somatic mtDNA deletions with ageing	ROS exposure + insufficient mitophagy → clonal expansion of mtDNA deletions in SN neurons	High proportion of ETC-deficient neurons (↓ COX activity), selective SN vulnerability	Human post-mortem single-cell sequencing: ↑ mtDNA deletions in PD vs. controls
Systemic mitochondrial vulnerability	Subtle bioenergetic deficits extend to peripheral cells	Potential biomarker utility → platelet CI activity, OXPHOS signatures, mtDNA CN variability	Peripheral assays; metabolomics, and proteomics studies
Mitochondrial dysfunction vs. oxidative stress (updated paradigm)	CI impairment → ↓ ATP and possible ROS changes; but: CI inhibition does not necessarily → pathological ROS ↑	ROS is not obligatorily linked to injury; toxicity is often due to loss of spare respiratory capacity rather than ROS overload	Rotenone work; CI-deficient mouse models lacking ROS elevation; failures of antioxidant trials
Redox signaling imbalance (not simple ROS excess)	Physiological ROS → signaling roles (mitochondrial biogenesis, plasticity); dysregulated redox signaling → neuronal vulnerability	Pathology driven by disrupted redox fidelity rather than absolute ROS levels	*In vitro* neuronal models; emerging *in vivo* redox biology
Convergent mitochondrial impairment as PD driver	CI dysfunction + mtDNA damage + impaired mitophagy → integrated bioenergetic decline → neuron-specific stress responses overwhelmed	Progressive SN degeneration; metabolic inflexibility	Multi-omics studies; human and animal data
Therapeutic implications	Stabilize CI assembly → enhance mitophagy (PINK1/Parkin pathways) → reduce pathological redox imbalance → support mitochondrial proteostasis	Potential disease-modifying strategies	Translational pipelines; biomarker development (respiration metrics, mtDNA instability, oxidative markers)

ATP, adenosine triphosphate; CI, complex I; CIV, complex IV; CN, copy number; COX, cytochrome c oxidase; DA, dopaminergic; DAT, dopamine transporter; ETC, electron transport chain; L-DOPA, levodopa; MPP^+^, 1-methyl-4-phenylpyridinium; MPTP, 1-methyl-4-phenyl-1,2,3,6-tetrahydropyridine; mtDNA, mitochondrial DNA; NADH, reduced nicotinamide adenine dinucleotide; OXPHOS, oxidative phosphorylation; PD, Parkinson’s disease; ROS, reactive oxygen species; SN, substantia nigra; TFAM, mitochondrial transcription factor A; VMAT2, vesicular monoamine transporter 2; ΔΨm, mitochondrial membrane potential; ↑, increase; ↓, decrease; →, leads to or contributes to.

Rotenone provides another important example of toxic PD converging on mitochondrial dysfunction. As a lipophilic pesticide, rotenone readily crosses biological membranes and directly inhibits mitochondrial complex I, reproducing several PD-relevant features in experimental models, including dopaminergic vulnerability, oxidative stress, impaired bioenergetics, and α-synuclein-related pathology. Importantly, this toxic mechanism overlaps with genetic forms of PD in which mutations in genes such as PINK1, PRKN, DJ-1, SNCA, LRRK2, and other PD-associated loci impair mitochondrial quality control, respiratory efficiency, organelle dynamics, trafficking, or redox homeostasis. Thus, toxic and genetic PD should not be considered mechanistically separate categories, but rather complementary routes toward a shared mitochondrial failure state. This convergence supports the view that mitochondria represent a central pathogenic node linking environmental complex I inhibitors, inherited defects in mitochondrial maintenance, and selective dopaminergic neurodegeneration (Lucchesi et al., 2025).

Epidemiological observations further support the relevance of complex I dysfunction to human disease. Chronic dietary exposure to annonaceous acetogenins—naturally occurring complex I inhibitors found in herbal preparations and tropical fruits—has been associated with atypical parkinsonism in parts of the Caribbean (Caparros-Lefebvre and Elbaz, 1999; Lannuzel et al., 2007). These findings reinforce the idea that environmental mitochondrial toxins can trigger parkinsonian syndromes in genetically susceptible individuals.

Post-mortem studies consistently report modest but significant reductions in complex I activity in the substantia nigra and frontal cortex of individuals with PD, accompanied by oxidative damage to both nuclear- and mitochondrial-encoded complex I subunits (Keeney et al., 2006; Parker et al., 2008). More recent proteomic and metabolomic analyses corroborate these findings, demonstrating disrupted assembly of oxidative phosphorylation (OXPHOS) complexes and increased oxidative stress in vulnerable neuronal populations (Grunewald et al., 2019). Peripheral tissues have also been investigated for evidence of systemic mitochondrial impairment. Early studies reported reduced platelet complex I activity in some patients with PD, but the finding was not sufficiently consistent or reproducible across cohorts and assay platforms to support diagnostic use. It is therefore best regarded as historical evidence of possible systemic bioenergetic dysfunction rather than a validated peripheral biomarker (Schapira and Jenner, 2011).

Experimental models provide mechanistic insights into how mild respiratory deficits affect dopamine homeostasis. Selective disruption of complex I function in dopaminergic neurons, such as targeted deletion of the Ndufs4 subunit, results in reduced dopaminergic release, increased dopamine turnover, and impaired vesicular storage, changes attributed to adenosine triphosphate (ATP) depletion and enhanced cytosolic dopamine metabolism (Choi et al., 2008; Sterky et al., 2011). These studies suggest that bioenergetic compromise may precipitate early dopaminergic dysfunction before overt neuronal loss.

### 2.2 Metabolic rewiring from OXPHOS to glycolysis as a resilience mechanism

An additional layer of mitochondrial resilience in PD may involve adaptive metabolic rewiring from OXPHOS toward glycolysis. In the classical view, neurons are considered highly dependent on mitochondrial respiration because of their large energetic requirements for membrane excitability, synaptic transmission, axonal transport, and neurotransmitter recycling. However, this view is being refined by evidence that neuronal metabolism is spatially compartmentalized and that not all neuronal domains depend on OXPHOS to the same extent. In particular, neuronal somata appear to preferentially use aerobic glycolysis rather than mitochondrial respiration, whereas nerve terminals show greater dependence on OXPHOS. This metabolic arrangement may be protective because it reduces mitochondrial oxygen consumption and electron leakage, thereby limiting reactive oxygen species production and preserving antioxidant capacity. Consistent with this idea, deletion of *Pkm2* shifted neuronal somata from aerobic glycolysis toward OXPHOS, increased oxidative damage, and promoted progressive dopaminergic neuronal loss, suggesting that somatic aerobic glycolysis may be intrinsically neuroprotective in vulnerable neuronal populations (Wei et al., 2023).

A similar protective principle has been observed in glial–neuronal metabolic interactions. Astrocytes are highly glycolytic cells and can provide metabolic support to neurons, in part through lactate production and transfer. In experimental models of traumatic injury, glycolytic preconditioning in astrocytes mitigated dopaminergic neurodegeneration through a Warburg-like shift involving mitochondrial redox signaling, HIF-1α activation, and reduced pyruvate entry into mitochondrial oxidation. These findings support the concept that glycolytic reallocation in astrocytes can buffer neuronal vulnerability under stress and that metabolic resilience may emerge not only from neuronal intrinsic mechanisms but also from coordinated astrocyte–neuron metabolic coupling (Solano Fonseca et al., 2021). In PD models, this concept is further supported by evidence that enhancement of glycolysis through activation of phosphoglycerate kinase 1 (PGK1), the first ATP-generating enzyme in the glycolytic pathway, increases ATP availability and attenuates disease progression. PGK1 activation by terazosin increased glycolytic activity, improved ATP levels, and reduced dopaminergic neurodegeneration in toxin-induced, genetic, and patient-derived PD models, with additional supportive associations in clinical databases (Cai et al., 2019). Thus, glycolysis may reflect an adaptive response that supports ATP production while limiting mitochondrial oxidative stress.

This glycolytic shift may also intersect with α-synuclein biology. Although aggregated or mislocalized α-synuclein can impair mitochondrial respiration, disrupt complex I activity, alter mitochondrial dynamics, and increase oxidative stress, soluble α-synuclein may also participate in context-dependent metabolic regulation. α-Synuclein has been shown to promote microglial glycolysis and migration through a PKM2-dependent mechanism, indicating that α-synuclein can directly influence glucose metabolism in non-neuronal cells involved in PD-related neuroinflammation (Qiao et al., 2019). More recently, a non-peer-reviewed preprint reported that α-synuclein interacts with lactate dehydrogenase A, increases lactate production and glycolytic flux, reduces mitochondrial reactive oxygen species, and supports resistance to subsequent complex I inhibition after low-dose rotenone preconditioning. These findings were interpreted as evidence of an α-synuclein-dependent mitohormetic response (Geula et al., 2025). Although this observation requires confirmation, it is consistent with the broader concept that α-synuclein may have dual, context-dependent effects, contributing to toxicity when aggregated or pathologically accumulated, but potentially supporting metabolic adaptation under selected stress conditions.

Hypoxia provides another paradigm in which reduced reliance on OXPHOS and activation of glycolytic programs may protect against mitochondrial stress. Under low oxygen availability, stabilization of hypoxia-inducible factors promotes transcriptional programs that reduce mitochondrial oxygen consumption and enhance glycolysis, including increased expression of glycolytic enzymes and lactate dehydrogenase A. In models of mitochondrial respiratory chain disease, genetic or pharmacological activation of the hypoxia response protected against mitochondrial toxicity, and chronic hypoxia improved survival, body weight, behavior, neuropathology, and disease biomarkers in a mouse model of Leigh syndrome (Jain et al., 2016). More recently, small-molecule induction of tissue hypoxia by increasing oxygen–hemoglobin affinity reproduced several benefits of hypoxia therapy in mitochondrial disease models, suggesting that hypoxia-like metabolic states may be pharmacologically tractable (Blume et al., 2025). These observations are relevant to PD because dopaminergic neurons are particularly vulnerable to mitochondrial oxygen stress, complex I impairment, and oxidative damage.

In PD-relevant models, chronic exposure to 11% oxygen prevented α-synuclein preformed fibril-induced dopaminergic neurodegeneration and motor impairment. Importantly, delayed initiation of hypoxia also halted further neuronal loss and improved motor dysfunction despite persistence of α-synuclein pathology, indicating that hypoxia may protect downstream of aggregate formation or independently of aggregate clearance (Marutani et al., 2025). This is conceptually important because it suggests that reducing oxygen-associated mitochondrial stress or activating HIF-1α-dependent glycolytic adaptation may mitigate neuronal vulnerability even when α-synuclein pathology is already present. These findings suggest that metabolic rewiring toward glycolysis, whether induced by neuronal compartmentalization, astrocytic support, PGK1 activation, α-synuclein-dependent metabolic modulation, or hypoxia/HIF-1α signaling, may reduce the toxic burden of mitochondrial oxygen metabolism and represent an underexplored component of mitochondrial resilience in PD.

### 2.3 Mitochondrial DNA damage and defective genomic maintenance

Mitochondrial oxidative phosphorylation relies on proteins encoded by both nuclear and mitochondrial genomes. Mitochondrial DNA (mtDNA) encodes 13 essential OXPHOS subunits, and its integrity is preserved by a specialized replication and repair system. Mutations in mtDNA can be inherited or acquired somatically, especially in long-lived, metabolically active cells such as neurons (Alston et al., 2017).

Experimental evidence indicates that defective mtDNA maintenance contributes to aging phenotypes and neurodegeneration. Mice with proofreading-deficient POLG mutations accumulate mtDNA errors, develop premature aging features, and display progressive respiratory chain defects (Trifunovic et al., 2004; Kujoth et al., 2005). In humans, variants in POLG1 are associated with parkinsonism, further supporting a link between impaired mtDNA fidelity and dopaminergic vulnerability (Rahman and Copeland, 2019).

Direct evidence for the role of mtDNA damage in PD comes from models in which mtDNA integrity is experimentally disrupted. Dopaminergic neuron–specific deletion of the mitochondrial transcription factor A (TFAM), which is essential for mtDNA transcription and replication, leads to progressive motor impairment, nigral neuron loss, and responsiveness to levodopa, mirroring key clinical features of PD (Ekstrand et al., 2007). Similarly, expression of mitochondrially targeted nucleases that induce mtDNA double-strand breaks causes selective degeneration of midbrain dopaminergic neurons (Pickrell et al., 2016).

Human post-mortem analyses show age-related accumulation of somatic mtDNA deletions in substantia nigra dopaminergic neurons, often reaching levels sufficient to impair cytochrome c oxidase activity (Bender et al., 2006; Kraytsberg et al., 2006). Single-cell sequencing studies have confirmed these findings and demonstrated that mtDNA deletions are more frequent in PD than in age-matched controls (Reeve et al., 2018; Taylor et al., 2022). Notably, these mutations are clonally expanded within individual neurons, indicating gradual accumulation over a lifetime rather than an inherited origin. Comparatively spared neuronal populations, including cortical and hippocampal pyramidal neurons, have substantially fewer mtDNA deletions, highlighting the selective vulnerability of substantia nigra neurons (Reeve et al., 2009).

Although current evidence does not support mtDNA mutations as a primary cause of sporadic PD, their accumulation likely contributes to disease progression (Kirches, 2009). Increased oxidative stress, impaired mitophagy, and reduced fidelity of mitochondrial quality-control pathways may accelerate mtDNA damage in vulnerable neurons (Wen et al., 2025). Because complex I contains several mtDNA-encoded subunits, it is particularly susceptible to the effects of accumulating mtDNA lesions.

These findings underscore a central paradigm: sporadic PD arises not from a single mitochondrial defect, but from convergent impairments in respiratory chain function, mtDNA maintenance, and cellular stress responses. These mechanisms are increasingly integrated into biomarker development, such as measures of mitochondrial respiration in peripheral tissues, mtDNA copy-number instability, or circulating markers of oxidative stress, and offer promising avenues for early disease detection (Borsche et al., 2021; Wu et al., 2023).

From a therapeutic perspective, strategies aimed at restoring mitochondrial quality control, enhancing mitophagy, stabilizing OXPHOS assembly, or reducing oxidative burden are gaining traction in translational research (Hu D. et al., 2021). As mechanistic understanding deepens, mitochondria are emerging not only as contributors to PD pathogenesis but also as promising targets for disease-modifying interventions.

### 2.4 Oxidative stress and its amplification in dopaminergic neurons

The prevailing model linking mitochondrial dysfunction to neurodegeneration in PD proposes that reduced complex I activity decreases ATP production and increases reactive oxygen species (ROS) production, initiating a self-amplifying cycle of oxidative damage and respiratory failure (Subramaniam and Chesselet, 2013). Early studies suggested that this cascade could explain the selective vulnerability of substantia nigra dopaminergic neurons, which operate under high metabolic demand and have relatively limited antioxidant defenses (Santana-Roman et al., 2025). Mitochondria are both a major source and as a primary target of ROS, placing them at the center of redox-related injury in multiple neurodegenerative disorders.

However, more recent experimental and translational evidence has challenged the long-standing assumption that mitochondrial dysfunction necessarily leads to pathological ROS accumulation *in vivo* (Chen et al., 2024). Several contemporary studies have shown that defects in oxidative phosphorylation can occur without a corresponding increase in oxidative stress, suggesting that mitochondrial failure and ROS overproduction are not obligatorily linked (Nicoletti et al., 2021). Neuronal susceptibility to complex I inhibition appears to be determined primarily by the loss of spare respiratory capacity rather than by excessive ROS generation, underscoring the central role of bioenergetic reserve in maintaining neuronal viability under metabolic stress (Yadava and Nicholls, 2007; Brand and Nicholls, 2011). Although rotenone elevates mitochondrial superoxide levels, scavenging superoxide does not reliably mitigate neuronal injury, highlighting a more nuanced relationship between respiratory chain impairment and redox imbalance (Siena et al., 2026).

Mouse models with severe defects in respiratory chain complexes reinforce this perspective. Despite profound bioenergetic deficits and increased susceptibility to apoptosis, these animals often show no measurable increase in ROS production or oxidative stress markers in affected tissues (Choi et al., 2008; Kolesar et al., 2019). These findings suggest that mitochondrial dysfunction can drive cell death independently of oxidative stress mechanisms, at least under specific experimental conditions.

At the same time, the role of ROS in cellular physiology has been re-evaluated (Zarkovic, 2020). Rather than being exclusively harmful, ROS participate in essential signaling pathways that regulate neuronal plasticity, mitochondrial biogenesis, and adaptive stress responses (Angelova and Abramov, 2018; Sies and Jones, 2020). A growing body of research highlights the importance of redox signaling fidelity—not simply increasing or decreasing ROS levels—in maintaining neuronal resilience (Li B. et al., 2025). Disruption of this balance, rather than ROS themselves, may better explain PD vulnerability.

**FIGURE 1:**
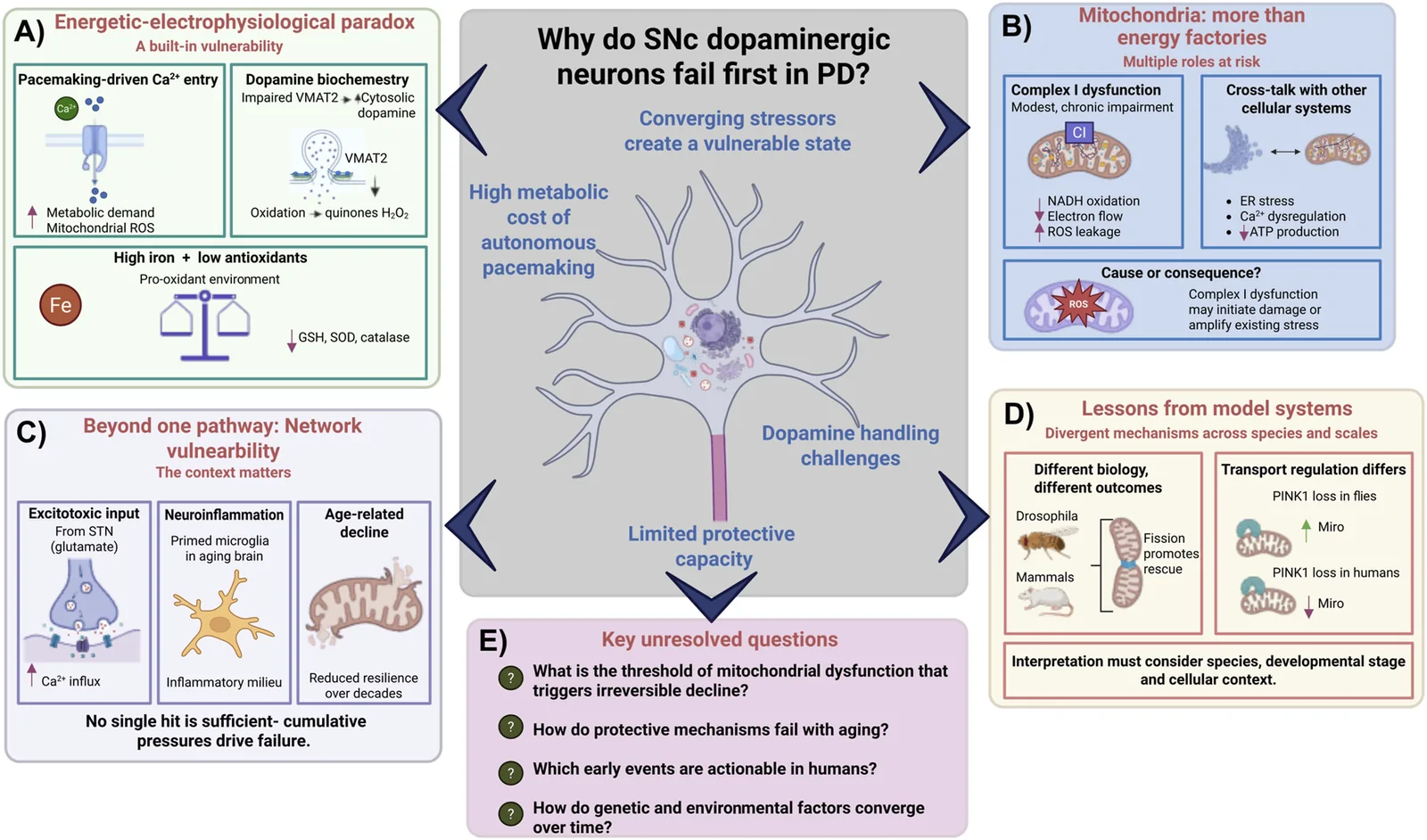
Convergent mitochondrial mechanisms underlying selective dopaminergic vulnerability in Parkinson’s disease. Environmental toxins, aging-related mitochondrial DNA damage, and genetic susceptibility converge on mitochondrial dysfunction in Parkinson’s disease. **(A)** Triggers include complex I inhibitors such as MPTP/MPP^+^ and annonaceous acetogenins, accumulation of somatic mitochondrial DNA mutations, and impaired mitochondrial DNA maintenance linked to POLG variants or TFAM dysfunction. **(B)** Mitochondrial dysfunction involves reduced complex I activity, defective oxidative phosphorylation assembly, mitochondrial DNA deletions, and decreased respiratory capacity, resulting in bioenergetic failure. Reactive oxygen species are not obligatorily increased, supporting a context-dependent role of redox imbalance. **(C)** Dual mechanistic output includes a primary bioenergetic pathway, characterized by reduced ATP production, impaired vesicular dopamine storage, cytosolic dopamine metabolism, and synaptic dysfunction, together with a modulatory redox pathway that may promote oxidative damage under specific conditions. **(D)** Failure of mitochondrial homeostasis results from impaired mitophagy and accumulation of dysfunctional mitochondria, creating a self-amplifying damage loop. **(E)** Selective vulnerability arises because substantia nigra pars compacta dopaminergic neurons have high metabolic demand, limited bioenergetic reserve, and progressive dopaminergic dysregulation, ultimately leading to neurodegeneration and motor dysfunction. ATP, adenosine triphosphate; BBB, blood–brain barrier; MPP^+^, 1-methyl-4-phenylpyridinium; MPTP, 1-methyl-4-phenyl-1,2,3,6-tetrahydropyridine; mtDNA, mitochondrial DNA; OXPHOS, oxidative phosphorylation; POLG, mitochondrial DNA polymerase gamma; ROS, reactive oxygen species; SNc, substantia nigra pars compacta; TFAM, mitochondrial transcription factor A; ↓, decreased or reduced; →, leads to or contributes to.

These insights call for a refined paradigm in which mitochondrial dysfunction, redox biology, and neuronal susceptibility are viewed as dynamically interconnected rather than linearly causal. For clinicians and neuroscientists, the implications are substantial. Biomarker development must move beyond global measures of oxidative stress toward more specific indicators of mitochondrial capacity, redox signaling, and metabolic adaptation. Therapeutic strategies should similarly shift from broad antioxidant approaches—which have repeatedly failed in clinical trials—to interventions that restore mitochondrial efficiency, stabilize complex I function, and preserve physiological ROS signaling. The convergence of environmental, aging-related, and genetic mitochondrial stressors on bioenergetic failure, impaired redox homeostasis, defective mitophagy, and selective dopaminergic vulnerability is summarized in Figure 1.

This evolving framework underscores the need to revisit entrenched assumptions about PD pathogenesis and encourages the development of mechanistically grounded, disease-modifying therapies that better reflect our current understanding of mitochondrial biology.

## 3 Mitochondrial consequences of Parkinson’s disease–associated genetic variants

### 3.1 Parkin: a multifunctional, stress-responsive E3 ubiquitin ligase in neuroprotection

The discovery of *PRKN* mutations in autosomal recessive parkinsonism in 1998 marked a turning point in PD genetics, revealing that disruptions in ubiquitin-mediated proteostasis profoundly affect dopaminergic neuron survival (Hattori and Mizuno, 2017). *PRKN* encodes parkin, a 465–amino acid cytosolic protein with an N-terminal ubiquitin-like (UBL) domain and a distinctive RBR (RING-between-RING) catalytic module (Clausen et al., 2024). This module consists of RING1, IBR, and RING2 elements, which together coordinate multiple zinc ions essential for proper structural folding. High-resolution structural analyses have also identified an additional RING0 domain that stabilizes zinc binding and participates in autoinhibition (Trempe et al., 2013).

These zinc-coordinating domains confer exceptional structural sensitivity. Pathogenic *PRKN* mutations often disrupt zinc binding, leading to protein misfolding and loss of function (Yi et al., 2019). In addition, the cysteine-rich structure of parkin makes it susceptible to irreversible inactivation under severe oxidative stress, a vulnerability supported by the presence of oxidatively modified, misfolded parkin species in the brains of individuals with sporadic PD (Wagner et al., 2025). Aberrant activation of c-Abl kinase, observed in dopaminergic neurons in PD, further impairs parkin activity by phosphorylating inhibitory sites, promoting its inactivation and aggregation (Karuppagounder et al., 2014).

Parkin acts as an E3 ubiquitin ligase, catalyzing mono- and polyubiquitination with diverse linkage types, thereby determining the fate of its substrates. Biochemical studies have clarified that parkin operates through a hybrid RING–HECT mechanism: RING1 binds the ubiquitin-conjugating enzyme E2, which then transfers ubiquitin to a conserved active-site cysteine in RING2 before final transfer to substrate lysines (Wenzel et al., 2011; Sauve et al., 2022). This dual mechanism provides the flexibility necessary for parkin to participate in a wide range of proteostatic and signaling functions.

More than 30 putative parkin substrates have been identified to date, spanning roles in mitochondrial dynamics, endoplasmic reticulum (ER) stress responses, vesicular trafficking, and apoptotic regulation (Kamienieva et al., 2021). These functions do not form a single linear pathway; rather, they highlight parkin’s broad capacity to maintain cellular homeostasis under diverse stress conditions.

A consistent theme across cell and animal studies is parkin’s potent cytoprotective capacity. Increased parkin expression protects neurons from mitochondrial injury, ER stress, excitotoxicity, and proteotoxicity (Wu et al., 2023). Conversely, loss of parkin sensitizes cells to multiple stressors, though germline *Prkn* knockout mice show surprisingly limited dopaminergic degeneration, an observation now attributed to developmental compensation. Conditional adult-onset *Prkn* deletion induces progressive nigral degeneration, affirming the crucial role of parkin in mature neurons (Frank-Cannon et al., 2008; Menon et al., 2024).

Parkin expression is dynamically regulated by stress. The transcription factors ATF4 and p53 enhance *PRKN* transcription, whereas c-Jun and N-myc act as repressors, integrating parkin into broader adaptive stress networks (Bouman et al., 2011). These regulatory dynamics position parkin as a central effector within integrated cellular defense programs.

One of the most influential advances in the past decade is the elucidation of the parkin–PARIS–PGC-1α axis. Parkin ubiquitinates and promotes the degradation of PARIS, a transcriptional repressor of PGC-1α (peroxisome proliferator-activated receptor gamma coactivator 1-alpha), the master co-activator of mitochondrial biogenesis and oxidative metabolism. Loss of parkin leads to accumulation of PARIS, suppression of PGC-1α activity, and impaired mitochondrial biogenesis (Stevens et al., 2015; Scott et al., 2022). Conditional deletion studies show that inhibiting PARIS can rescue dopaminergic neuron survival in *Prkn*-deficient mice, highlighting this transcriptional circuit as a promising therapeutic target.

The evolving understanding of parkin positions it not simply as a genetic cause of early-onset PD, but as a central coordinator of mitochondrial quality control, stress adaptation, and neuronal resilience (McCoy and Cookson, 2012). This broader perspective has several translational implications: a) Disease mechanisms: Sporadic and familial PD converge on shared pathways involving impaired mitophagy, disrupted proteostasis, and defective stress signaling. b) Biomarker development: Circulating or tissue-based markers of parkin activity, PARIS accumulation, or PGC-1α signaling may inform early diagnosis or therapeutic monitoring. c) Therapeutics: Strategies to enhance parkin activity, stabilize its active conformation, inhibit its oxidative or kinase-mediated inactivation, or modulate downstream transcriptional programs are emerging as plausible disease-modifying approaches.

These insights exemplify the field’s shift from a narrow focus on dopaminergic neuron loss to an integrated view of mitochondrial homeostasis, genome–environment interactions, and dynamic stress responses. Understanding parkin within this multidimensional framework will be essential for developing future neuroprotective therapies.

#### 3.1.1 Parkin at the intersection of neurodegeneration and cancer

The identification of *PRKN* within the highly unstable FRA6E fragile site on chromosome 6q25–q27 first suggested that parkin might have roles beyond neuroprotection (Cesari et al., 2003). Common fragile sites are genomic regions prone to replication stress, chromosomal breakage, and structural rearrangements, and are increasingly recognized as contributors to oncogenesis. Subsequent genomic profiling of breast, ovarian, lung, and hepatocellular carcinomas revealed recurrent deletions or reduced expression of *PRKN*, positioning parkin as a putative tumor suppressor whose loss may facilitate malignant transformation (Ikeuchi et al., 2019; Ahn et al., 2020).

Experimental evidence accumulated over the past decade strongly supports this tumor suppressor role. In murine models, heterozygous *Prkn* deletion accelerates adenoma formation in the presence of mutant *APC*, implicating parkin in Wnt-regulated intestinal tumorigenesis. Similarly, *Prkn* knockout mice show increased susceptibility to irradiation-induced lymphomagenesis, and certain lines develop spontaneous hepatic tumors marked by excessive hepatocyte proliferation (Wu et al., 2025). Restoration of parkin expression in parkin-deficient cancer cell lines, such as glioma or lung carcinoma, reduces tumorigenicity in xenograft models, although effects on basal proliferation vary across tumor contexts (Park et al., 2019).

Despite robust genetic and functional evidence, the molecular basis of parkin’s anticancer activity remains incompletely defined. Early observations that parkin ubiquitinates and promotes the degradation of cyclin E—including within neuronal cells—suggested that loss of parkin may permit aberrant G1–S phase progression and genomic instability (Um et al., 2006). Elevated cyclin E levels have been identified in several parkin-deficient tumors, although this association is not universal, underscoring the heterogeneity of parkin-associated tumorigenic pathways (Tay et al., 2010).

More recent advances highlight parkin’s broader influence on cellular metabolism. Cancer cells are characterized by a shift from mitochondrial oxidative phosphorylation toward aerobic glycolysis—the Warburg effect—which supports rapid proliferation despite reduced ATP efficiency (Liberti and Locasale, 2016). Parkin has emerged as a transcriptional target and effector of p53, a key regulator of metabolic homeostasis. Loss of parkin disrupts p53-dependent transcriptional programs governing mitochondrial respiration and antioxidant defense, thereby enhancing glycolytic flux and reinforcing the metabolic phenotype characteristic of malignant cells (Du et al., 2025). In both human lung cancer cells and mouse embryonic fibroblasts, parkin deficiency reduces mitochondrial respiration and increases glycolytic dependence, directly linking parkin loss to tumor-promoting metabolic reprogramming (Du et al., 2025).

Parkin also influences lipid metabolism, an increasingly recognized aspect of metabolic adaptation in cancer. In mice fed a high-fat, high-cholesterol diet, *Prkn* expression is strongly upregulated, which stabilizes the fatty acid transporter CD36 and increases lipid uptake (Kim et al., 2011). Notably, *Prkn* knockout mice are resistant to diet-induced weight gain and hepatic insulin resistance, highlighting parkin´s role in integrating lipid homeostasis with metabolic stress responses (Kim et al., 2011).

Whether germline *PRKN* mutations increase cancer susceptibility in humans remains unresolved (Schule et al., 2015). The rarity of parkin-linked parkinsonism and the need for large, well-characterized cohorts have hindered definitive epidemiological analyses. Recent studies indicate no major increase in cancer risk among individuals with heterozygous *PRKN* variants, though subtle effects cannot be excluded (Blauwendraat et al., 2020; Lesage et al., 2020).

The convergence of mechanistic pathways underlying neurodegeneration and tumorigenesis has important translational implications. Parkin acts as a regulator of genomic stability, metabolic integrity, and cellular resilience, functions that, when disrupted, predispose neurons to degeneration and proliferating cells to malignant transformation. This duality underscores the need for a more integrative framework that considers how stress-response pathways influence both neuroprotective and oncogenic outcomes.

From a therapeutic perspective, understanding the shared mechanisms by which parkin maintains mitochondrial health, controls proteostasis, and modulates cell-cycle regulators may inform strategies to enhance neuronal survival in PD and suppress tumor growth in certain cancers. As research continues to clarify the metabolic and transcriptional circuits regulated by parkin, these insights are likely to yield new biomarkers and therapeutic targets in both neurology and oncology.

### 3.2 PINK1 — A mitochondrial kinase integrating quality control, bioenergetics, and neuronal vulnerability

The identification of *PINK1* as a causative gene for autosomal recessive early-onset PD in 2004 established a decisive link between mitochondrial homeostasis and selective dopaminergic neurodegeneration (Valente et al., 2004). *PINK1* encodes a 581–amino acid serine/threonine kinase with an N-terminal mitochondrial targeting sequence, a transmembrane helix, and a highly conserved kinase domain. More than 30 pathogenic variants have since been reported, most of which reduce kinase activity, destabilize the protein, or impair mitochondrial targeting, consistent with a loss-of-function mechanism (Kondapalli et al., 2012; Trinh et al., 2014).

The subcellular localization of PINK1 has been extensively debated. Early studies reported PINK1 at the outer and inner mitochondrial membranes and in the cytosol, creating uncertainty about its physiological distribution (Qiao et al., 2024). However, a unified model has emerged in which PINK1 undergoes tightly regulated import and processing that depend on mitochondrial membrane potential (*Δψm*).

Under basal conditions, PINK1 is imported through the TOM and TIM23 complexes into mitochondria, where sequential cleavage by mitochondrial processing peptidase and the inner-membrane rhomboid protease PARL generates a short, unstable PINK1 fragment that is rapidly degraded in the cytosol (Greene et al., 2012; Trempe et al., 2013; Trempe and Gehring, 2023). This continuous turnover keeps PINK1 levels low when mitochondria are healthy. When *Δψm* collapses, import and PARL-mediated proteolysis are abruptly inhibited, allowing full-length PINK1 to accumulate at the outer mitochondrial membrane. There, PINK1 dimerizes and autophosphorylates, initiating the recruitment and activation of parkin, a critical trigger for mitophagy (Bayne and Trempe, 2019; Trempe and Gehring, 2023). Association with the TOM complex on depolarized mitochondria is thought to facilitate rapid PINK1 re-import and pathway termination once membrane potential is restored (Lazarou et al., 2012).

These findings clarify why PINK1 has been detected in multiple subcellular compartments: its localization reflects a dynamic quality-control system that distinguishes functional from dysfunctional mitochondria (Chu, 2010). PINK1 exerts broad cytoprotective effects across model systems. Loss of PINK1 in mice results in increased sensitivity to mitochondrial toxins such as MPTP, and these phenotypes can be rescued by overexpression of parkin or DJ-1, suggesting that these PD-associated proteins act in convergent or parallel pathways (Gispert et al., 2009).

Efforts to identify downstream effectors have revealed that PINK1 phosphorylates several mitochondrial and cytosolic proteins involved in bioenergetics, proteostasis, and stress adaptation. TRAP1, a mitochondrial chaperone, is one such substrate; its phosphorylation enhances resistance to oxidative stress (Pridgeon et al., 2007). PINK1 also interacts with the serine protease HtrA2/Omi, enhancing its stress-activated protease activity through phosphorylation and promoting mitochondrial resilience (Plun-Favreau et al., 2007).

A major conceptual advance in the field has been the recognition that disrupted mitochondrial motility is a critical step in mitophagy (Wang et al., 2026). PINK1 interacts with the outer-membrane GTPases MIRO1 and MIRO2 and with the adaptor protein MILTON/TRAK, which links mitochondria to kinesin and dynein motors for axonal transport (Weihofen et al., 2009). Multiple studies now demonstrate that PINK1 phosphorylates MIRO1, marking it for parkin-dependent proteasomal degradation. Degradation of MIRO1 detaches mitochondria from microtubule motors, halts their movement, and enables damaged mitochondria to be sequestered and cleared via mitophagy (Hsieh et al., 2016; Shlevkov et al., 2016). Notably, species-specific differences have been observed: PINK1 loss increases MIRO abundance in *Drosophila* but reduces MIRO levels in mammalian cells, underscoring divergent compensatory responses across taxa.

PINK1 also modulates mitochondrial trafficking through its interaction with MARK2, a kinase that regulates microtubule dynamics. Truncated PINK1 variants promote anterograde transport, while full-length PINK1 enhances retrograde movement, indicating context-dependent roles in coordinating mitochondrial positioning relative to neuronal metabolic demands (Lu et al., 2023).

The protective functions of PINK1 extend beyond mitophagy. In multiple model systems, PINK1 deficiency leads to fragmented mitochondrial networks, reduced membrane potential, and impaired respiratory chain function—particularly complex I activity—as well as increased susceptibility to energy failure under high-demand conditions (Morais et al., 2009). These defects contribute to synaptic dysfunction, as reserve-pool synaptic vesicle mobilization depends on adequate ATP supply.

Disrupted calcium homeostasis is another mechanism linking PINK1 loss to neuronal injury. PINK1-deficient neurons, especially those derived from human pluripotent stem cells, exhibit mitochondrial calcium overload in response to cellular stress due to dysfunctional Na^+^/Ca^2+^ exchange (Gandhi et al., 2009). Calcium overload increases ROS production, inhibits glucose uptake, and precipitates respiratory decline, creating a feed-forward cycle that may accelerate dopaminergic degeneration (Gandhi et al., 2009).

Recent advances have redefined PINK1 from a single-function kinase to a central regulator of mitochondrial fate, transport, metabolism, and calcium handling (Bayne and Trempe, 2019). For clinicians and neuroscientists, this evolving understanding has several implications: a) Diagnosis and early detection: Biomarkers of impaired mitophagy, altered PINK1 processing, or MIRO1 degradation may serve as early indicators of mitochondrial dysfunction before overt neurodegeneration. b) Therapeutic development: Small molecules that stabilize PINK1, enhance its kinase activity, or mimic its phosphorylation of parkin, MIRO1, or TRAP1 are promising targets for disease-modifying therapy. c) Conceptual shift: PD should increasingly be viewed as a disorder of integrated mitochondrial quality control rather than a primary defect of dopaminergic neurons.

As knowledge of the PINK1 regulatory network continues to expand, it is clear that therapeutic strategies aimed at restoring mitochondrial integrity, rather than simply increasing dopamine replacement, may offer the most promising path to neuroprotection in PD.

### 3.3 Coordinated PINK1-Parkin signaling in the maintenance of mitochondrial integrity

Although neither *Prkn* nor *Pink1* knockout mice display overt neurodegeneration or motor impairment under basal conditions, extensive research indicates that this apparent normalcy results from developmental compensation rather than true redundancy (Watzlawik et al., 2024). In multiple cellular models, loss of either parkin or PINK1 increases susceptibility to mitochondrial stress, reduces the capacity to maintain membrane potential, and impairs recovery after toxic injury (Barodia et al., 2017). These findings suggest that the PINK1–parkin axis is dispensable under resting conditions but becomes essential when mitochondrial integrity is compromised.

*Drosophila* studies have been particularly influential in clarifying the hierarchy of these pathways. Both *parkin* and *pink1* mutants exhibit remarkably similar phenotypes, including shortened lifespan, male sterility, defective flight muscles, and severe mitochondrial dysfunction (Park et al., 2025). The observation that parkin expression rescues *pink1* deficiency, but not *vice versa*, supports a linear pathway in which PINK1 acts upstream to activate parkin (Pickrell and Youle, 2015).

The seminal work of Narendra and colleagues established that parkin is selectively recruited to depolarized mitochondria in a PINK1-dependent manner, initiating mitophagy, the selective autophagic removal of damaged mitochondria (Narendra D. P. et al., 2010). Chemical dissipation of mitochondrial membrane potential, such as with carbonyl cyanide m-chlorophenyl hydrazone (CCCP), leads to rapid PINK1 accumulation at the outer mitochondrial membrane. This localization is both necessary and sufficient to recruit parkin, even when PINK1 is artificially targeted to other organelles such as peroxisomes or lysosomes (Lazarou et al., 2012). These findings established PINK1 as the molecular sensor of mitochondrial injury and parkin as the downstream effector that labels damaged organelles for destruction.

Mitophagy depends on the coordinated action of numerous cofactors that link ubiquitinated mitochondrial proteins to the autophagic machinery (Ding and Yin, 2012). Although p62/SQSTM1 was initially proposed to be essential for parkin-mediated mitophagy, later studies showed that its primary role is to promote perinuclear clustering rather than mitophagic degradation itself (Narendra D. et al., 2010). Additional adaptors—including NDP52, OPTN, TAX1BP1, and TBK1—have now emerged as more central regulators of mitophagy in mammalian cells (Lazarou et al., 2012). Other modulators, such as HDAC6 and AMBRA1, facilitate autophagosome–lysosome fusion or phagophore initiation, highlighting the multistep nature of the pathway.

A major advance in recent years has come from quantitative proteomics, which shows that PINK1 activation triggers widespread ubiquitination of outer mitochondrial membrane proteins (Trempe and Gehring, 2023). Parkin catalyzes both Lys48-and Lys63-linked ubiquitin chains, supporting parallel functions: proteasomal degradation of selected substrates and recruitment of autophagic adaptors (Ordureau et al., 2018). Among the most strongly ubiquitinated substrates are mitofusin one and 2 (MFN1/2), the GTPases MIRO1/2, and components of the TOM import machinery (Gegg et al., 2010). Degradation of MFN proteins favors mitochondrial fission, which facilitates segregation of damaged organelles, while ubiquitination of MIRO arrests mitochondrial movement along microtubules, allowing injured mitochondria to be isolated from the healthy network (Shlevkov et al., 2016).

Proteasomal degradation is required for efficient mitophagy. Blocking the proteasome prevents the initial remodeling of the outer membrane, thereby halting subsequent autophagosome formation (Lu et al., 2022). Although several parkin substrates have been identified, no single target appears strictly required for mitophagy, suggesting that broad ubiquitin remodeling—rather than modification of one specific protein—is the critical signal for organelle clearance (Uoselis et al., 2023).

**FIGURE 2:**
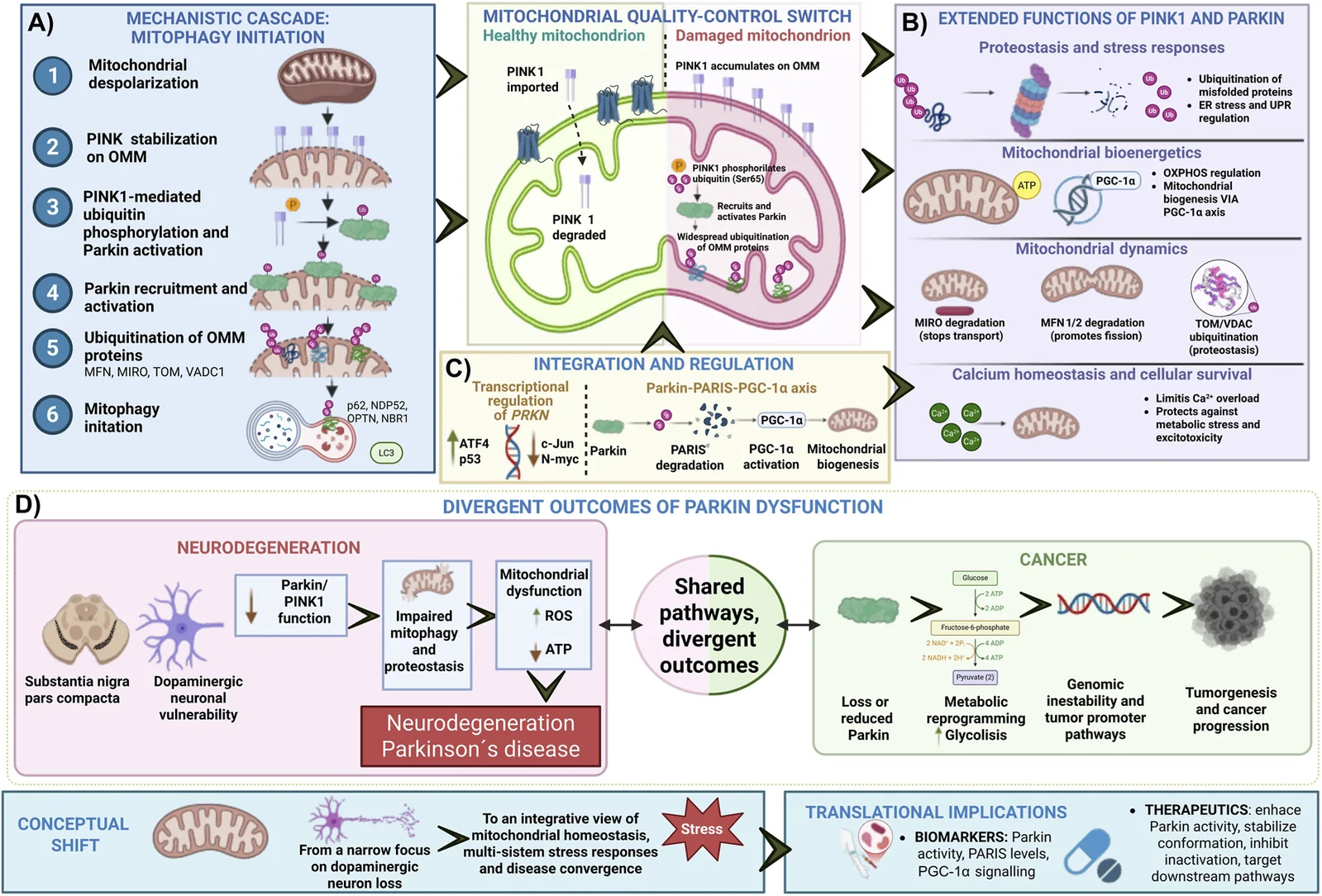
The PINK1–Parkin axis as a mitochondrial quality-control system linking mitochondrial integrity to disease outcomes. The PINK1–Parkin pathway coordinates mitochondrial surveillance, mitophagy, stress adaptation, and metabolic homeostasis. **(A)** Mitophagy initiation. Mitochondrial depolarization prevents normal PINK1 import and degradation, allowing PINK1 accumulation on the outer mitochondrial membrane. PINK1 phosphorylates ubiquitin at Ser65 and promotes Parkin recruitment and activation, leading to ubiquitination of outer mitochondrial membrane proteins, including MFN, MIRO, TOM, and VDAC1, followed by recruitment of autophagic adaptors and mitophagy initiation. **(B)** Extended functions. Beyond canonical mitophagy, PINK1 and Parkin contribute to proteostasis, endoplasmic reticulum stress responses, mitochondrial bioenergetics, mitochondrial dynamics, trafficking, calcium homeostasis, and cell survival. **(C)** Regulation and integration. Parkin expression is transcriptionally regulated by stress-responsive pathways, while Parkin-mediated PARIS degradation promotes PGC-1α activation and mitochondrial biogenesis. **(D)** Divergent outcomes of dysfunction. Impaired PINK1–Parkin signaling promotes defective mitophagy, mitochondrial dysfunction, ATP depletion, oxidative stress, and dopaminergic neurodegeneration. In proliferating cells, Parkin loss may also favor metabolic reprogramming, genomic instability, and tumorigenesis, illustrating shared stress-response pathways with divergent disease outcomes. ATP, adenosine triphosphate; Ca^2+^, calcium ion; ER, endoplasmic reticulum; LC3, microtubule-associated protein 1A/1B-light chain 3; MFN, mitofusin; MIRO, mitochondrial Rho GTPase; OMM, outer mitochondrial membrane; OXPHOS, oxidative phosphorylation; PARIS, Parkin-interacting substrate; PGC-1α, peroxisome proliferator-activated receptor gamma coactivator 1-alpha; PINK1, PTEN-induced kinase 1; ROS, reactive oxygen species; TOM, translocase of the outer mitochondrial membrane; Ub, ubiquitin; UPR, unfolded protein response; VDAC1, voltage-dependent anion channel 1.

Whether PINK1 directly phosphorylates parkin—and whether parkin ubiquitinates PINK1—remains an area of active investigation. Several studies support direct interaction and reciprocal modification, whereas others find no such evidence, reflecting methodological differences and the dynamic nature of the PINK1–parkin complex (Winklhofer and Haass, 2010). What is firmly established, however, is that PINK1 phosphorylates ubiquitin at Ser65, creating a feed-forward mechanism that dramatically enhances parkin activation (Kazlauskaite et al., 2014). The canonical mitophagy cascade, the mitochondrial quality-control switch, and the broader consequences of PINK1–Parkin dysfunction are summarized in Figure 2.

Recognition of the PINK1-parkin mitophagy pathway has fundamentally reshaped our understanding of Parkinson’s disease pathogenesis (Ward et al., 2024). Instead of viewing mitochondrial failure as a late-stage consequence of neurodegeneration, accumulating evidence indicates that impaired mitochondrial quality control is an early, potentially initiating event. This paradigm shift has significant implications: a) Diagnostic advances—circulating phosphorylated ubiquitin or mitophagy biomarkers may enable earlier detection of mitochondrial dysfunction; b) Therapeutic strategies—include small-molecule PINK1 activators, parkin stabilizers, and agents that enhance mitophagy adaptor recruitment represent emerging avenues for disease modification; c) Precision medicine—individuals with *PINK1* or *PRKN* mutations may benefit from targeted therapies designed to restore quality-control pathways. Table 2 summarizes the principal mitochondrial effects of Parkin and PINK1 and their relevance to mitochondrial quality control in Parkinson’s disease.

**TABLE 2:** Mitochondrial effects of PD-associated genes Parkin and PINK1.

Gene/Pathway	Key molecular features (→)	Mitochondrial consequences	Mechanistic evidence and models
Parkin (PRKN) — *E3 ligase governing mitochondrial proteostasis and stress adaptation*	UBL domain → RING0/RING1/IBR/RING2 → Zn^2+^ coordination → auto-inhibition relieved by PINK1; RING–HECT hybrid mechanism → E2 binding → transthiolation → substrate ubiquitination	Maintains mitochondrial integrity via: (i) broad ubiquitination of OMM proteins (K48/K63 chains) → proteasomal removal + mitophagy recruitment; (ii) regulation of mitochondrial dynamics/ER stress; (iii) control of metabolic programmes via PARIS–PGC-1α axis	PRKN mutations → disrupted Zn^2+^ binding, misfolding; parkin inactivated by oxidative stress and c-Abl phosphorylation; conditional adult Prkn loss → DA neuron degeneration; parkin overexpression protects against mitochondrial toxins
Parkin substrate network	>30 substrates: MFN1/2, MIRO1/2, TOM/TIM components, PARIS, ER–mitochondria contacts	MFN ubiquitination → fission-priming; MIRO ubiquitination → arrest of mitochondrial motility; PARIS degradation → ↑PGC-1α → ↑ mitochondrial biogenesis	Ubiquitinomics: widespread OMM ubiquitination upon parkin activation; PARIS inhibition rescues Prkn-deficient mice
Parkin regulation	Stress-responsive TFs: ATF4 and p53 → ↑ PRKN transcription; c-Jun/N-myc → repression; c-Abl → inhibitory phosphorylation	Integrates parkin into cellular stress networks; oxidative inhibition or kinase modification → loss of cytoprotection	Oxidized/misfolded parkin detected in sporadic PD; c-Abl inhibitors restore parkin activity
Parkin in cancer biology	PRKN in FRA6E fragile site → deletion-prone; parkin ubiquitinates cyclin E → control of G1–S; regulates p53 metabolic output → OXPHOS vs. glycolysis balance	Loss of parkin → ↑ genomic instability, ↑ glycolysis (Warburg shift), altered lipid uptake (CD36 stabilization)	Tumor models: ↑ adenomas (APC background), ↑ lymphomas, spontaneous hepatic tumors; re-expression → ↓ tumorigenicity
PINK1 — *Mitochondrial kinase that senses Δψm and triggers mitophagy*	MTS → TOM/TIM import → MPP/PARL cleavage; Δψm-dependent processing; loss-of-function mutations → impaired kinase activity or import	Basal turnover maintains low PINK1; depolarization → PINK1 stalling at OMM → dimerization → autophosphorylation → parkin recruitment/activation	PINK1 localization clarified by Δψm-dependent import blockade; pathogenic variants reduce stability or trafficking
PINK1 kinase targets	Ubiquitin (Ser65) → phospho-Ub → potent parkin activation; TRAP1 → ↑ oxidative-stress resistance; HtrA2 → ↑ stress protease activity; MIRO1 phosphorylation → degradation when parkin-engaged	Enhances mitoproteostasis: ↑ parkin activity; ↑ stress resilience; MIRO removal → arrest of mitochondrial movement → priming for mitophagy	Structural/biochemical studies: phospho-Ub feed-forward activation loop; MIRO phosphorylation conserved in stress signaling
PINK1 effects on mitochondrial dynamics and trafficking	PINK1–MIRO–TRAK/kinesin axis → motility arrest upon depolarization; MARK2 interactions → directionality control; truncated vs. full-length PINK1 → anterograde vs. retrograde bias	Prevents damaged mitochondria from propagating dysfunction; positions mitochondria according to metabolic needs	*Drosophila* vs. mammalian differences in MIRO turnover; trafficking defects precede bioenergetic failure
PINK1 loss-of-function phenotypes	↓ Δψm maintenance; ↓ CI activity; fragmented mitochondrial networks; impaired Ca^2+^ handling → mitochondrial Ca^2+^ overload → ↑ ROS, ↓ glucose utilization	Synaptic dysfunction (ATP-dependent vesicle mobilization); heightened toxin sensitivity (MPTP, rotenone); energy crisis under stress	*Drosophila*, mouse, and human iPSC-derived neurons show conserved deficits
Coordinated PINK1–Parkin axis — *Central mitochondrial quality-control module*	Δψm loss → PINK1 accumulation → Ub-Ser65 phosphorylation → parkin activation → widespread OMM ubiquitination → recruitment of NDP52/OPTN/TAX1BP1/TBK1 → autophagosome formation	Mitophagy: segregation and removal of damaged mitochondria; requires OMM remodeling, MFN degradation, MIRO clearance, proteasome priming; ensures mitochondrial network fidelity	CCCP models: PINK1 localization sufficient to recruit parkin even ectopically; proteomics: ∼hundreds of OMM ubiquitination sites; parkin rescue of pink1 mutants in *Drosophila* confirms pathway order
Mitophagy cofactors	p62 → clustering (not essential for degradation); NDP52/OPTN/TBK1 → core mitophagy effectors; HDAC6 → autophagosome–lysosome fusion; AMBRA1 → phagophore initiation	Efficient mitophagy requires an ubiquitin-dependent adaptor network; proteasome inhibition blocks early OMM remodeling	Mammalian cell studies; TBK1-linked PD mutations highlight pathway vulnerability
Functional implications for PD	Parkin/PINK1 impairment → defective mitophagy → accumulation of damaged mitochondria → ↑ vulnerability to stress → DA neuron injury	Mitochondrial QC failure emerges as an early pathogenic event, not a downstream “consequence”	iPSC neurons, knock-in models, human tissue phospho-Ub biomarkers
Translational relevance	Stabilize parkin → protect against oxidative and kinase inactivation; activate PINK1 → boost phospho-Ub signaling; enhance adaptor recruitment → improve mitophagy flux; target PARIS–PGC-1α axis → restore mitochondrial biogenesis	Potential disease-modifying strategies for PD; biomarkers: phospho-Ub, PINK1 processing, PARIS accumulation, mitophagy flux signatures	Clinical pipelines exploring PINK1 activators, c-Abl inhibitors, parkin stabilizers

AMBRA1, autophagy and beclin 1 regulator 1; ATF4, activating transcription factor 4; ATP, adenosine triphosphate; Ca^2+^, calcium ion; CCCP, carbonyl cyanide m-chlorophenyl hydrazone; CI, complex I; DA, dopaminergic; ER, endoplasmic reticulum; HDAC6, histone deacetylase 6; HECT, homologous to the E6-AP carboxyl terminus; HtrA2, high-temperature requirement protein A2; IBR, in-between-RING domain; iPSC, induced pluripotent stem cell; MARK2, microtubule affinity-regulating kinase 2; MFN1/2, mitofusin 1/2; MIRO1/2, mitochondrial Rho GTPase 1/2; MPP, mitochondrial processing peptidase; MPTP, 1-methyl-4-phenyl-1,2,3,6-tetrahydropyridine; MTS, mitochondrial targeting sequence; NDP52, nuclear dot protein 52; OMM, outer mitochondrial membrane; OPTN, optineurin; OXPHOS, oxidative phosphorylation; PARIS, Parkin-interacting substrate; PARL, presenilin-associated rhomboid-like protein; PD, Parkinson’s disease; PGC-1α, peroxisome proliferator-activated receptor gamma coactivator 1-alpha; PINK1, PTEN-induced kinase 1; PRKN, Parkin RBR E3 ubiquitin protein ligase; QC, quality control; RBR, RING-between-RING; RING, really interesting new gene domain; ROS, reactive oxygen species; TAX1BP1, Tax1-binding protein 1; TBK1, TANK-binding kinase 1; TFs, transcription factors; TIM, translocase of the inner mitochondrial membrane; TOM, translocase of the outer mitochondrial membrane; TRAK, trafficking kinesin-binding protein; TRAP1, tumor necrosis factor receptor-associated protein 1; Ub, ubiquitin; UBL, ubiquitin-like domain; Δψm, mitochondrial membrane potential; Zn^2+^, zinc ion; ↑, increased or enhanced; ↓, decreased or reduced; →, leads to, promotes, or contributes to.

As the field advances toward mechanism-based therapeutic development, the PINK1–parkin axis is among the most promising druggable pathways in neurodegeneration. Future work integrating structural biology, systems neuroscience, and clinical biomarker studies will be essential to translate these mechanistic insights into interventions that meaningfully alter the course of PD.

### 3.4 The pathophysiological significance of mitophagy in Parkinson’s disease

Genetic evidence strongly implicates defective mitophagy in PD: pathogenic mutations in *PINK1* and *PRKN* (parkin) disrupt key steps in mitochondrial quality control, undermining the cell’s ability to identify and clear dysfunctional mitochondria (Geisler et al., 2010; Iorio et al., 2022). In healthy cells, PINK1 functions as a sensor of mitochondrial damage, while parkin acts as the effector that tags impaired mitochondria for degradation via ubiquitination (Narendra and Youle, 2011).

However, early foundational studies often relied on non-physiological conditions—overexpression of PINK1 or parkin in immortalized tumor cell lines or fibroblasts treated acutely with protonophores (e.g., CCCP) or ionophores to depolarize mitochondria (Narendra and Youle, 2011; Mouton-Liger et al., 2017). These paradigms raised important questions about whether mitophagy occurs at endogenous expression levels, in response to physiologically relevant stress, and whether its impairment meaningfully contributes to neuronal loss *in vivo*.

More recent work in cellular and animal systems addresses these concerns, suggesting that PINK1–parkin–dependent mitophagy is engaged under relevant stressors—such as mtDNA mutations, bioenergetic challenge, or oxidative stress—but its activation appears tightly regulated, cell-type–specific, and context-dependent (Jin and Youle, 2012; Vincow et al., 2013). For example, in mitophagy-reporter mice, basal mitophagy is readily detectable in many peripheral tissues, yet appears limited in the brain under physiological conditions (McWilliams et al., 2018), suggesting that neurons may use alternative or more selective quality-control mechanisms.

Further evidence linking mitophagy failure to PD-relevant pathology has emerged from murine models combining parkin or PINK1 deficiency with additional mitochondrial stress (such as mutator mtDNA polymerase) (Vizziello et al., 2021). In these models, impaired mitophagy led to accumulation of damaged mitochondria, release of mtDNA, activation of innate immunity via the cGAS–STING pathway, neuroinflammation, and eventual dopaminergic neuron loss (Decout et al., 2021). This connects mitochondrial clearance directly to inflammation-induced neurodegeneration, broadening the pathophysiological framework beyond classical protein aggregation paradigms.

Therefore, current data support at least three central tenets: a) PINK1–parkin–mediated mitophagy is a *bona fide*, physiologically relevant quality-control pathway (Jin and Youle, 2012). It is activated under conditions of mitochondrial stress or high energetic demand, even if only at low basal levels in neurons (McWilliams et al., 2018). b) Neuronal mitophagy is stringently regulated, likely spatially and temporally restricted, especially in postmitotic, energy-demanding cells, to avoid compromising bioenergetic capacity (McWilliams et al., 2018). c) Impaired mitophagy may initiate or accelerate neurodegeneration through inflammation and bioenergetic failure, rather than as a bystander to protein aggregation. Loss of mitochondrial integrity may release damage-associated molecular patterns (DAMPs), such as mtDNA, triggering innate immune activation and contributing to neuronal demise (Newman and Shadel, 2018). Table 3 summarizes the pathophysiological relevance of mitophagy and mitochondrial quality control in Parkinson’s disease.

**TABLE 3:** Pathophysiological relevance of mitophagy and mitochondrial quality control in Parkinson’s disease.

Pathogenic node	Key molecular events (→)	Mitochondrial/cellular consequences	Mechanistic evidence and models
PINK1–Parkin–mediated mitophagy (core QC pathway)	Δψm loss → PINK1 import arrest → OMM accumulation → Ub-Ser65 phosphorylation → parkin recruitment → OMM ubiquitination (K48/K63) → adaptor engagement (NDP52/OPTN/TAX1BP1/TBK1) → mitophagosome formation	Clearance of dysfunctional mitochondria; suppression of mtDNA leakage; prevention of innate immune activation (cGAS–STING)	Pathogenic PINK1/PRKN mutations disrupt sensing or ubiquitination; mitophagy reporters show context-specific activation; parkin/PINK1 + mtDNA mutator mice: ↑ mtDNA release → neuroinflammation → DA neuron loss
Physiological relevance and limitations of early models	Overexpression systems + CCCP depolarization → non-physiological mitophagy induction	Raised doubts regarding endogenous pathway activity	Updated *in vivo* models: mitophagy occurs under stressors (mtDNA mutation, ROS, energetic load) and is cell-type and context dependent
Tissue-specific mitophagy patterns	Basal mitophagy is robust in peripheral tissues → limited in healthy brain; neuronal mitophagy spatially/temporally restricted	Neurons may rely on alternative QC pathways → heightened vulnerability when damaged mitochondria accumulate	Mitophagy reporter mice: low neuronal basal turnover; stress-induced mitophagy demonstrable but tightly controlled
Mitophagy failure → inflammation-driven degeneration	Impaired PINK1/parkin → damaged mitochondria persist → mtDNA release → cGAS–STING activation → IFN signaling → neuroinflammation	Mitochondrial DAMP-driven injury contributes to PD progression	Parkin/PINK1-deficient + mitochondrial-stress models: innate immune activation precedes nigral degeneration
Implications for PD pathogenesis	Mitophagy impairment → early, initiating defect (not downstream consequence of aggregation)	PD reframed as mitochondrial QC disorder	Biomarkers: phospho-Ub, ubiquitinated OMM proteins, inflammatory signatures
Therapeutic logic	Boost PINK1 stability/activity → enhance parkin activation → modulate adaptor recruitment; avoid indiscriminate mitophagy overactivation	Restore QC balance; prevent inflammatory sequelae	Clinical pipeline exploring targeted mitophagy modulation

CCCP, carbonyl cyanide m-chlorophenyl hydrazone; cGAS, cyclic GMP–AMP synthase; DA, dopaminergic; DAMP, damage-associated molecular pattern; IFN, interferon; K48/K63, lysine 48- and lysine 63-linked ubiquitin chains; mtDNA, mitochondrial DNA; NDP52, nuclear dot protein 52; OMM, outer mitochondrial membrane; OPTN, optineurin; PD, Parkinson’s disease; PINK1, PTEN-induced kinase 1; PRKN, Parkin RBR E3 ubiquitin protein ligase; QC, quality control; ROS, reactive oxygen species; Ser65, serine 65; STING, stimulator of interferon genes; TAX1BP1, Tax1-binding protein 1; TBK1, TANK-binding kinase 1; Ub, ubiquitin; Δψm, mitochondrial membrane potential; ↑, increased or enhanced; →, leads to, promotes, or contributes to.

For clinicians and neuroscientists, this evolving view requires a conceptual shift: PD should be regarded not only as a synucleinopathy but also as a disorder of mitochondrial quality-control homeostasis. Consequently, therapeutic strategies must move beyond symptomatic dopamine replacement and protein-aggregation inhibitors; instead, they should aim to restore or fine-tune mitophagy, preserve mitochondrial integrity, and prevent inflammation. Biomarkers of mitophagy flux—such as phospho-ubiquitin species, ubiquitinated mitochondrial proteins, or downstream inflammation signals—may hold promise for early diagnosis and for monitoring disease-modifying interventions.

### 3.5 Mitochondrial dynamics: fission–fusion balance within the broader quality-control network

Mitochondria are dynamic, highly networked organelles that continually undergo fusion, fission, trafficking, and turnover, processes essential for meeting metabolic demand, regulating calcium, and responding to stress (Greene et al., 2012). The balance among these processes is critical; dysregulation is implicated in various neurodegenerative diseases.

Fusion is mediated primarily by the mitofusins MFN1 and MFN2 (outer membrane) and OPA1 (inner membrane), while fission depends on cytosolic GTPases such as DRP1, with additional regulation by accessory factors (Adebayo et al., 2021). Mitophagy integrates with these dynamics, coordinating structural remodeling and selective degradation of damaged organelles (Ma et al., 2020).

In mammalian cells, including neurons, acute loss of PINK1 or parkin often causes DRP1-dependent mitochondrial fragmentation, reduced membrane potential, and diminished ATP production (Lutz et al., 2009). These changes can be ameliorated by promoting fusion (for example, overexpressing MFN2 or OPA1) or inhibiting fission (for example, using dominant-negative DRP1), indicating that altered dynamics may precede or contribute to energetic failure.

However, data from non-mammalian models (e.g., *Drosophila*) have yielded apparently contradictory results: in flies, *pink1* or *parkin* deficiency causes neuromuscular degeneration, which is paradoxically rescued by increasing mitochondrial fission or reducing fusion (Deng et al., 2008). These differences likely arise from species-specific compensatory strategies, differences in metabolic demand and turnover, or distinct relative contributions of mitophagy versus fission–fusion dynamics.

Recent mechanistic studies underscore that PINK1 and parkin modulate mitochondrial architecture in a context-dependent manner. Under mild stress, parkin may ubiquitinate pro-fission proteins (e.g., DRP1 or FIS1) to stabilize the network and preserve mitochondrial function. Under severe damage, however, parkin favors ubiquitination and degradation of outer membrane fusion proteins such as MFN1/2, and mitochondrial–ER contact tethers such as MFN2, promoting fission, isolation, and mitophagy (McLelland et al., 2018).

This dual functionality—network stabilization under low stress and quality-control isolation under high stress—aligns with an adaptive model in which PINK1–parkin activity helps balance energy production, organelle integrity, and cellular survival. Crucially, these processes appear to be disrupted in PD, particularly under sustained or repeated mitochondrial insults, such as aging, environmental toxins, or metabolic stress (Yamada et al., 2025).

For translational neuroscience, this broader conception of mitochondrial quality control has two major implications. First, therapeutic interventions should not aim solely to boost mitophagy indiscriminately; rather, they should modulate mitochondrial dynamics adaptively, promoting fusion and network integrity under mild stress, but allowing fission and clearance when damage is irreparable. Second, combining assessments of mitochondrial dynamics, mitophagy flux, and bioenergetics may yield a more accurate picture of neuronal vulnerability and therapeutic response than any single parameter. Table 4 summarizes the fission–fusion–trafficking axis and its relevance to mitochondrial quality control in Parkinson’s disease.

**TABLE 4:** Mitochondrial dynamics and network remodeling in PD (fission–fusion–trafficking axis).

Process	Key molecular events (→)	Consequences for mitochondria and neurons	Evidence and context
Fusion	MFN1/2 (OMM) + OPA1 (IMM) → network connectivity → content mixing	Maintains Δψm, ATP production, and Ca^2+^ buffering	Altered in PD models; MFN2 anxiety in PINK1/parkin loss
Fission	DRP1 recruitment → constriction → division	Enables segregation of damaged mitochondria; supports mitophagy	Acute PINK1/parkin loss → DRP1-dependent fragmentation
Stress-dependent parkin functions	Mild stress → parkin ubiquitinates pro-fission proteins (e.g., DRP1, FIS1) → network stabilization; severe stress → MFN1/2 ubiquitination → fission and isolation	Dual mode: maintain bioenergetic continuity vs. prepare mitochondria for mitophagy	McLelland et al. (2018) and Iorio et al. (2022)
Species-specific compensation	*Drosophila*: pink1/parkin deficiency → degeneration rescued by ↑ fission or ↓ fusion	Indicates divergent QC priorities across taxa	Neuromuscular phenotypes in flies vs. mammalian neurons
Translational implications	Adaptive modulation of dynamics rather than blanket activation of fusion/fission	Better preservation of metabolic capacity while maintaining QC	Biomarkers: MFN/OPA1/DRP1 phosphorylation ratios; mtDNA release

ATP, adenosine triphosphate; Ca^2+^, calcium ion; DRP1, dynamin-related protein 1; FIS1, mitochondrial fission 1 protein; IMM, inner mitochondrial membrane; MFN1/2, mitofusin 1/2; mtDNA, mitochondrial DNA; OMM, outer mitochondrial membrane; OPA1, optic atrophy protein 1; PD, Parkinson’s disease; PINK1, PTEN-induced kinase 1; QC, quality control; Δψm, mitochondrial membrane potential; ↑, increased or enhanced; ↓, decreased or reduced; →, leads to, promotes, or contributes to.

**FIGURE 3:**
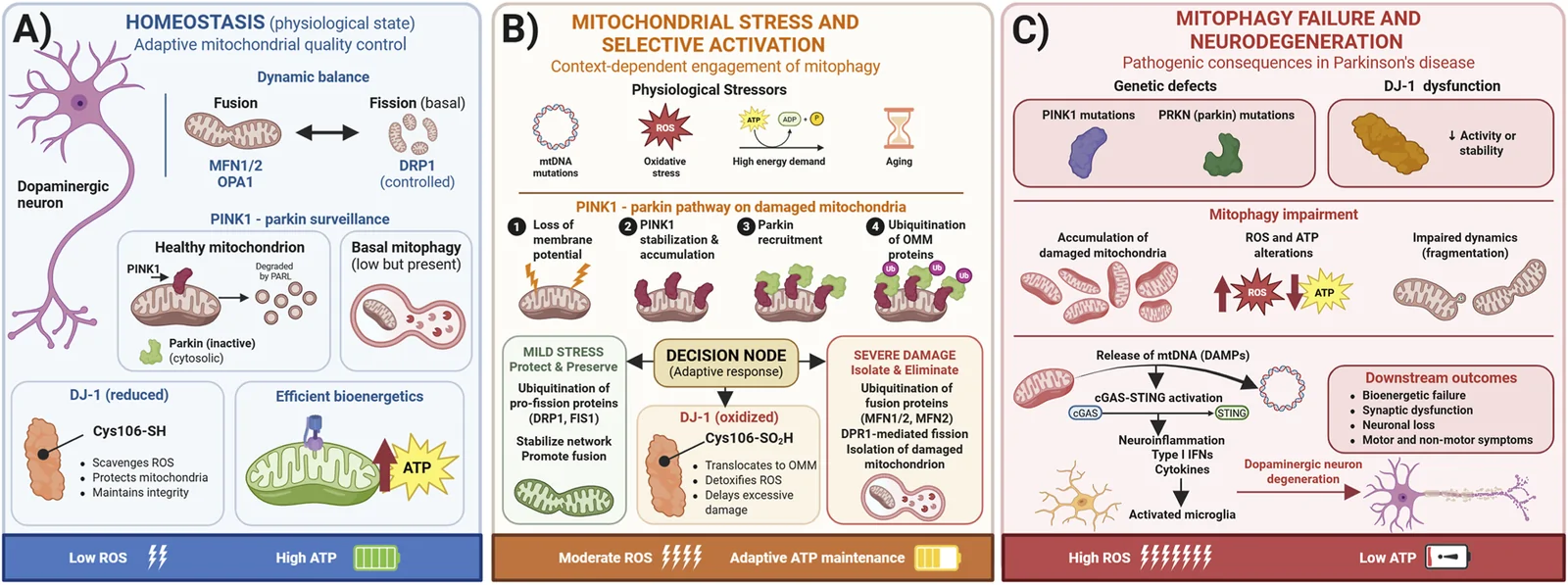
Mitochondrial quality control in Parkinson’s disease: from adaptive homeostasis to mitophagy failure and neurodegeneration. Mitochondrial quality control supports dopaminergic neuron survival by coordinating mitochondrial dynamics, PINK1–Parkin surveillance, basal mitophagy, redox buffering, and bioenergetic stability. **(A)** Homeostasis. In physiological conditions, balanced fusion and basal fission, regulated mainly by MFN1/2, OPA1, and DRP1, preserve mitochondrial network integrity. Healthy mitochondria import and degrade PINK1, while Parkin remains inactive in the cytosol. Low-level basal mitophagy supports organelle turnover, and reduced DJ-1 helps limit reactive oxygen species and maintain ATP production. **(B)** Mitochondrial stress and adaptive activation. Mitochondrial DNA mutations, oxidative stress, high energetic demand, and aging promote loss of membrane potential, PINK1 stabilization on the outer mitochondrial membrane, Parkin recruitment, and ubiquitination of outer mitochondrial membrane proteins. Depending on damage severity, this response may preserve the mitochondrial network or promote DRP1-dependent fission and selective elimination of damaged mitochondria. Oxidized DJ-1 contributes to redox buffering. **(C)** Mitophagy failure. Dysfunction of PINK1, PRKN/Parkin, or DJ-1 impairs mitochondrial quality control, leading to damaged mitochondria accumulation, fragmentation, ATP depletion, oxidative stress, and mitochondrial DNA release. These damage signals activate cGAS–STING-dependent innate immune pathways, promoting neuroinflammation, microglial activation, synaptic dysfunction, dopaminergic neurodegeneration, and motor and non-motor manifestations of Parkinson’s disease. ATP, adenosine triphosphate; cGAS, cyclic GMP–AMP synthase; Cys106, cysteine 106; DAMPs, damage-associated molecular patterns; DRP1, dynamin-related protein 1; FIS1, mitochondrial fission 1 protein; IFNs, interferons; MFN1/2, mitofusin 1/2; mtDNA, mitochondrial DNA; OMM, outer mitochondrial membrane; OPA1, optic atrophy protein 1; PINK1, PTEN-induced kinase 1; PRKN, Parkin RBR E3 ubiquitin protein ligase; ROS, reactive oxygen species; STING, stimulator of interferon genes; Ub, ubiquitin.

Emerging data reveals a more complex and nuanced understanding of PD pathogenesis. Rather than a straightforward cascade from mitochondrial dysfunction to ROS generation to cell death, PD may arise from failures in a tightly regulated mitochondrial quality-control network that encompasses mitophagy, dynamics, transport, bioenergetics, and immune signaling (Figure 3).

This paradigm shift has practical implications. It suggests that: a) Early biomarkers of dysfunctional mitophagy or maladaptive mitochondrial dynamics (such as phospho-ubiquitin, altered fusion/fission protein ratios, or indicators of mtDNA release) may identify individuals at risk before overt neurodegeneration; b) Therapeutic strategies should aim for homeostatic modulation rather than indiscriminate overactivation: restoring balance in mitochondrial turnover, dynamics, and signaling according to cellular context and stress load; and c) Clinical trials could benefit from incorporating mitochondrial and inflammatory biomarkers—not just α-synuclein load or dopaminergic function—as endpoints.

In summary, viewing PD as a disorder of mitochondrial homeostasis, rather than solely a proteinopathy, opens new translational avenues, requires rethinking diagnostic criteria, and emphasizes preventing neurodegeneration by maintaining mitochondrial and cellular resilience.

### 3.6 DJ-1 as a redox-sensitive guardian of mitochondrial homeostasis

Loss-of-function mutations in *PARK7*, the gene encoding DJ-1, are a rare but informative cause of autosomal recessive early-onset parkinsonism (Bonifati et al., 2003). DJ-1 is a ubiquitously expressed 189-amino-acid protein in a conserved superfamily that includes the bacterial stress-inducible chaperone Hsp31. Structural studies show that DJ-1 forms a stable homodimer, with an extensive dimer interface essential for its functional conformation (Bason et al., 2015).

Although DJ-1 has been attributed with diverse functions, consistent evidence identifies it as a critical redox-responsive protein. A conserved cysteine residue at position 106 undergoes rapid and reversible oxidation to cysteine-sulfinic acid in the presence of ROS, serving as the primary sensor and effector of DJ-1’s cytoprotective activity (van der Reest et al., 2018). Oxidation of Cys106 produces a characteristic acidic shift in DJ-1’s isoelectric point and enhances its stability and chaperone-like activity. Mutations that disrupt this cysteine abolish DJ-1’s protective function, underscoring its central role in oxidative stress responses.

DJ-1 knockout mice do not develop spontaneous nigrostriatal degeneration, but they show impaired scavenging of mitochondrial hydrogen peroxide, consistent with proposed peroxiredoxin-like peroxidase activity (Lopert and Patel, 2014). Under basal conditions, DJ-1 is primarily cytosolic, but oxidative or mitochondrial stress induces its translocation to the outer mitochondrial membrane, where it helps maintain mitochondrial integrity (Junn et al., 2009).

DJ-1 loss in neurons, patient-derived fibroblasts, and cultured cells leads to mitochondrial fragmentation, membrane depolarization, and increased ROS production, phenotypes that can be reversed by antioxidants or by re-expression of parkin or PINK1 (McCoy and Cookson, 2011). In contrast, DJ-1 cannot rescue the mitochondrial phenotypes associated with PINK1 or parkin deficiency, suggesting that DJ-1 acts in a parallel, partially compensatory pathway rather than upstream or downstream of the PINK1–parkin axis (Thomas et al., 2011).

Importantly, mitochondria from DJ-1–deficient brain or muscle tissues show increased ROS production even without overt neurodegeneration, indicating that DJ-1 supports mitochondrial resilience long before cell death pathways are activated (Andres-Mateos et al., 2007; De Lazzari et al., 2023). This is consistent with the broader understanding that PD-related genetic lesions often produce subtle, stress-dependent phenotypes that require additional metabolic, inflammatory, or environmental insults to manifest clinically.

Although early hypotheses suggested a physical interaction or complex formation between DJ-1, parkin, and PINK1, subsequent studies using size-exclusion chromatography and proteomics have not supported the existence of a stable trimeric complex (Thomas et al., 2011). Instead, DJ-1 appears to intersect with mitochondrial quality control by modulating redox homeostasis, thereby indirectly influencing pathways regulated by PINK1 and parkin (Thomas et al., 2011; Trempe et al., 2013; Trempe and Gehring, 2023).

DJ-1 increases neuronal resistance to mitochondrial toxins, reduces stress-induced mitochondrial fragmentation, and attenuates oxidative injury, functional parallels to PINK1 and parkin but achieved through distinct biochemical mechanisms (Krebiehl et al., 2010). Unlike PINK1 or parkin, DJ-1 is not required for canonical mitophagy signaling. Instead, DJ-1 acts as an upstream buffer against oxidative stress, preventing mitochondrial depolarization and a ROS surge that would otherwise trigger mitophagy or apoptosis in susceptible neurons (Im et al., 2010).

These observations highlight that DJ-1 represents an independent arm of mitochondrial defense, a redox-protective pathway that operates in parallel with, but not subordinate to, PINK1–parkin mitophagy (Thomas et al., 2011). Combined with evidence linking DJ-1 dysfunction to metabolic alterations, inflammation, and impaired mitochondrial calcium handling, this positions DJ-1 as a nodal regulator of neuronal stress resilience (Jha et al., 2017). Table 5 summarizes the role of DJ-1 as a redox-responsive mitochondrial protector in Parkinson’s disease.

**TABLE 5:** DJ-1 (PARK7) — redox-responsive mitochondrial protector.

Molecular feature	Key mechanistic steps (→)	Mitochondrial outcomes	Evidence and models
Redox-sensing architecture	Cys106 oxidation → Cys-SO_2_H formation → structural stabilization → enhanced chaperone-like activity	Limits ROS surge; prevents mitochondrial depolarization; increases stress resilience	DJ-1 KO mice: impaired mitochondrial H_2_O_2_ scavenging; acidic pI shift of oxidized DJ-1
Stress-induced mitochondrial recruitment	ROS/mitochondrial stress → DJ-1 translocation to OMM	Preserves morphology; constrains fragmentation; supports Δψm	Observed in neurons and fibroblasts; reversed by antioxidants
Loss-of-function phenotypes	DJ-1 deficiency → fragmentation → ↓ Δψm → ↑ ROS → Ca^2+^ handling defects	Sets vulnerability threshold prior to overt degeneration	Rescue: parkin or PINK1 overexpression (but not *vice versa*)
Relationship to PINK1/parkin	Parallel, partially compensatory pathway (not upstream/downstream)	Redox buffering indirectly influences the mitophagy threshold	No stable trimeric complex with PINK1/parkin (proteomics)
Clinical/therapeutic implications	Oxidized DJ-1 isoforms as biomarkers; stabilization of redox-active cysteine; small-molecule activators	Long-term neuroprotection via redox homeostasis	Relevant to sporadic and familial PD risk modulation

Ca^2+^, calcium ion; Cys106, cysteine 106; Cys-SO_2_H, cysteine-sulfinic acid; DJ-1, Parkinsonism-associated deglycase encoded by PARK7; H_2_O_2_, hydrogen peroxide; KO, knockout; OMM, outer mitochondrial membrane; PARK7, gene encoding DJ-1; PD, Parkinson’s disease; pI, isoelectric point; PINK1, PTEN-induced kinase 1; ROS, reactive oxygen species; Δψm, mitochondrial membrane potential; ↑, increased or enhanced; ↓, decreased or reduced; →, leads to, promotes, or contributes to.

The evolving understanding of DJ-1 biology has several implications for clinical neurology and therapeutic innovation: a) biomarkers of oxidative stress, including Cys106-oxidized DJ-1 isoforms, may serve as early indicators of mitochondrial dysfunction in PD or prodromal states; b) therapeutic strategies targeting DJ-1 stabilization, modulation of its redox-active cysteine, or pharmacological enhancement of its peroxidase-like activity are being explored as potential disease-modifying approaches; c) disease heterogeneity in PD may reflect differential vulnerability among mitochondrial quality-control pathways, with DJ-1 deficiency priming neurons for damage that manifests clinically only in combination with PINK1- or parkin-mediated impairments.

These insights underscore the need for a paradigm shift that frames PD not solely as a synucleinopathy but as a multifaceted disorder of mitochondrial resilience, redox homeostasis, and cellular stress adaptation.

### 3.7 α-synuclein: structural plasticity linking genetic and sporadic forms of Parkinson’s disease

α-Synuclein occupies a pivotal position in current models of PD pathogenesis, bridging monogenic and sporadic forms of the disorder. After its initial identification as a cause of autosomal-dominant PD, large genome-wide association studies have repeatedly confirmed *SNCA* as one of the strongest genetic risk loci for sporadic PD (Grover et al., 2022; Kim et al., 2024; Arias-Carrion et al., 2026). This convergence of rare, high-penetrance mutations and common risk alleles underscores α-synuclein’s central role in disease initiation and progression.

α-Synuclein is a 140–amino-acid presynaptic protein composed of three regions: an N-terminal amphipathic domain with imperfect 11-mer repeats that adopt α-helical structure upon membrane association; a hydrophobic, β-aggregation–prone NAC region; and an acidic C-terminal tail rich in regulatory motifs (Uéda et al., 1993; Davidson et al., 1998). This modular organization confers remarkable conformational flexibility. In aqueous solution, the protein behaves largely as an intrinsically disordered monomer, yet it can transition into membrane-bound helices, soluble oligomers, protofibrils, and amyloid fibrils (Davidson et al., 1998; Conway et al., 2000; Arias-Carrion et al., 2025).

The existence of physiological multimers—particularly helical tetramers—remains debated. Early studies suggested that endogenous α-synuclein forms stable tetramers that resist aggregation, whereas PD-linked mutations shift the equilibrium toward monomeric, aggregation-prone species (Dettmer et al., 2015). Subsequent work, however, found α-synuclein predominantly monomeric in human, murine, and cultured cell extracts (Smaldone et al., 2015; Mor et al., 2019). A synthesis of these data favors a dynamic equilibrium in which transient multimeric species exist but can be readily destabilized, making shifts in this conformational landscape a central determinant of pathology (Gurry et al., 2013; Fernandez and Lucas, 2018).

Importantly, converging experimental evidence indicates that small, soluble oligomers—not mature fibrils—are the principal neurotoxic species. These prefibrillar intermediates disrupt membranes, impair synaptic vesicle cycling, and compromise mitochondrial homeostasis (Calabresi et al., 2023; Arias-Carrion et al., 2025). This insight has catalyzed a shift in therapeutic strategy toward stabilizing physiological multimers or preventing toxic oligomer formation.

Despite extensive investigation, the full physiological repertoire of α-synuclein remains incompletely defined (Calabresi et al., 2023; Arias-Carrion et al., 2025). Its presynaptic localization and lipid-binding properties suggest involvement in vesicle trafficking and synaptic plasticity (Maroteaux et al., 1988; Arias-Carrion et al., 2025; Treviño et al., 2025). Biochemical studies show that α-synuclein promotes SNARE complex assembly by simultaneously binding to phospholipids and synaptobrevin-2, ensuring efficient synaptic vesicle fusion during neurotransmission (Treviño et al., 2025).

Mouse models indicate that α-synuclein modulates, rather than enables, synaptic transmission. Deletion of α-synuclein—or all three synucleins—enhances stimulus-evoked dopamine release, supporting a physiological role in constraining vesicle mobilization (Burré et al., 2010; Anwar et al., 2011; Treviño et al., 2025). Conversely, mild overexpression analogous to *SNCA* duplication or triplication reduces the synaptic vesicle recycling pool and diminishes neurotransmitter release in cultured neurons and *in vivo* (Nemani et al., 2010; Soll et al., 2020; Roman-Vendrell et al., 2025).

These findings support a model in which α-synuclein fine-tunes synaptic vesicle availability and release probability (Treviño et al., 2025). Both loss of function and toxic gain of function can produce early synaptic dysfunction, likely preceding structural neurodegeneration by years. Recent work links α-synuclein to mitochondrial impairment, a defining feature of PD pathology (Malpartida et al., 2021). α-Synuclein localizes to mitochondrial membranes and interacts directly with cardiolipin, a phospholipid critical for organization of the respiratory chain (Gilmozzi et al., 2020). Under conditions of overexpression or conformational instability, α-synuclein disrupts mitochondrial morphology, impairs oxidative phosphorylation, and increases susceptibility to oxidative stress (Geibl et al., 2024). Table 6 summarizes the structural plasticity of α-synuclein and its interface with mitochondrial and synaptic dysfunction in Parkinson’s disease.

**TABLE 6:** α-Synuclein—structural plasticity and mitochondrial interface.

Structural features	Mechanistic actions (→)	Mitochondrial and synaptic consequences	Evidence and models
Modular domains: N-terminal amphipathic helix + NAC (aggregation-prone) + acidic C-terminus	Membrane binding → curvature sensing; SNARE chaperoning; oligomer formation	At physiological levels, it regulates vesicle availability; at pathological levels, it oligomerizes and induces membrane disruption	Synaptic release phenotypes in knockout or overexpression models
Oligomerization vs. fibrils	Shift toward soluble toxic oligomers → membrane permeabilization → Ca^2+^ dysregulation	Early synaptic and mitochondrial dysfunction precede neuron death	Toxicity rescued by chaperones (e.g., TRAP1)
Mitochondrial interaction	Binding to cardiolipin → altered cristae; fragmentation without full depolarization	↓ OXPHOS; ↑ sensitivity to oxidative stress	Mitochondrial fragmentation in α-syn models is independent of Δψm collapse
Propagation mechanisms	Misfolded α-syn → secretion (vesicles/exosomes) → uptake → templated seeding → circuit spread	Progressive involvement of interconnected networks	Fetal graft pathology; fibril inoculation models
QC cross-talk	PINK1/parkin deficiency modifies α-syn toxicity; α-syn overload stresses QC pathways	Aggregation ↔ mitochondrial dysfunction reinforcing loop	Parkin KO delays A30P degeneration; mitophagy modulation rescues α-syn phenotypes
Therapeutic implications	Stabilize multimers, inhibit oligomerization, block spread, restore mitochondrial resilience	Targets early pathogenic steps before irreversible degeneration	Biomarkers: conformers, oligomers, organellar stress signatures

A30P, alanine-to-proline substitution at position 30 in α-synuclein; Ca^2+^, calcium ion; KO, knockout; NAC, non-amyloid-β component region; OXPHOS, oxidative phosphorylation; PD, Parkinson’s disease; PINK1, PTEN-induced kinase 1; QC, quality control; SNARE, soluble N-ethylmaleimide-sensitive factor attachment protein receptor; TRAP1, tumor necrosis factor receptor-associated protein 1; α-syn, α-synuclein; Δψm, mitochondrial membrane potential; ↑, increased or enhanced; ↓, decreased or reduced; →, leads to, promotes, or contributes to; ↔, bidirectional or mutually reinforcing interaction.

Notably, mitochondrial fragmentation associated with α-synuclein accumulation occurs even without of clear depolarization or overt toxicity (Nakamura, 2013). These early disturbances in organellar architecture may prime neurons for subsequent energetic failure. Manipulation of mitochondrial quality-control pathways can mitigate α-synuclein toxicity: the mitochondrial chaperone TRAP1 attenuates α-synuclein-induced damage (Butler et al., 2012), while mice lacking parkin show delayed degeneration in the context of A30P α-synuclein overexpression (Casadei et al., 2014). These findings reinforce the concept that PD arises from a maladaptive interplay between protein aggregation and mitochondrial quality-control failure.

One of the most influential insights of the past 2 decades is the recognition that α-synuclein pathology can propagate between cells, driving disease spread across interconnected neural circuits (Kordower et al., 2008; Volpicelli-Daley et al., 2011; Arias-Carrion et al., 2025). Clinical evidence comes from PD patients who received fetal midbrain grafts and later developed Lewy pathology within the transplanted neurons (Kordower et al., 2008; Li et al., 2008). Experimental models have shown that α-synuclein can be secreted via exocytosis and exosomes, taken up by endocytosis or passive diffusion, and seed endogenous α-synuclein in recipient neurons (Emmanouilidou et al., 2010; Alvarez-Erviti et al., 2011).

Synthetic α-synuclein fibrils or brain homogenates from transgenic animals induce robust aggregation and neurodegeneration when injected into naïve mice, even in regions distant from the injection site (Volpicelli-Daley et al., 2011; Luk et al., 2012). These findings place α-synuclein alongside tau, Aβ, and huntingtin in a broader class of misfolded, self-propagating proteins and provide a mechanistic rationale for early, circuit-targeted immunotherapies.

**FIGURE 4:**
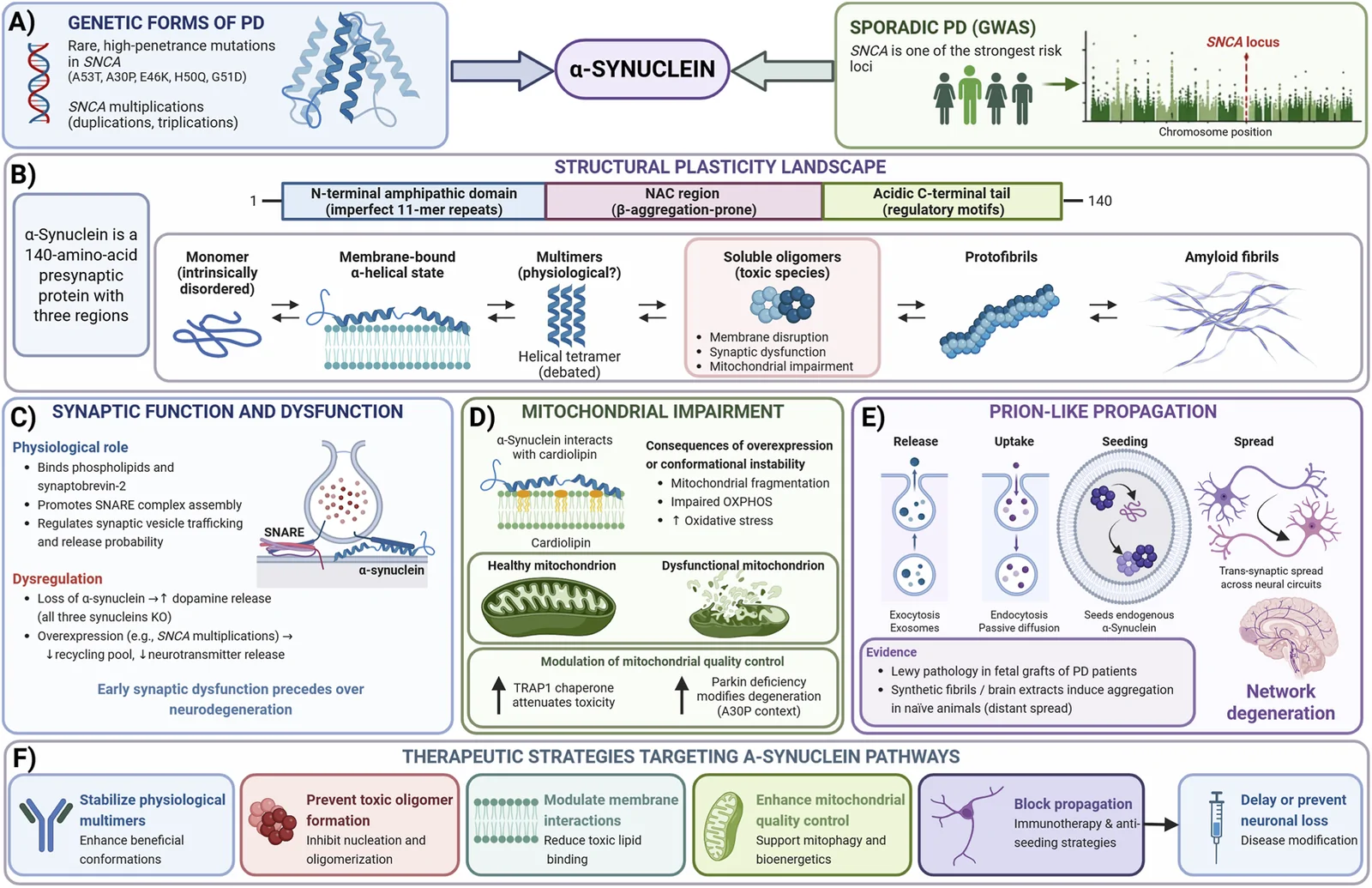
α-Synuclein structural plasticity links genetic susceptibility, mitochondrial dysfunction, and neurodegeneration in Parkinson’s disease. α-Synuclein acts as a central pathogenic hub connecting genetic risk, synaptic dysfunction, mitochondrial impairment, intercellular propagation, and therapeutic development. **(A)** Genetic contribution. Rare high-penetrance SNCA mutations and SNCA multiplications cause familial Parkinson’s disease, whereas genome-wide association studies identify the SNCA locus as a major susceptibility signal in sporadic disease. **(B)** Structural plasticity. α-Synuclein is a 140-amino-acid presynaptic protein with an N-terminal amphipathic domain, an aggregation-prone NAC region, and an acidic C-terminal tail. It can shift among intrinsically disordered monomers, membrane-bound α-helical states, putative multimers, soluble oligomers, protofibrils, and amyloid fibrils. Toxic oligomeric species are associated with membrane disruption, synaptic dysfunction, and mitochondrial injury. **(C)** Synaptic function. Physiologically, α-synuclein supports SNARE complex assembly and regulates synaptic vesicle trafficking; dysregulation contributes to early synaptic failure. **(D)** Mitochondrial impairment. Pathogenic α-synuclein species interact with cardiolipin, promote mitochondrial fragmentation, impair oxidative phosphorylation, and increase oxidative stress. **(E)** Propagation. Misfolded α-synuclein can be released, taken up by neighboring cells, seed endogenous α-synuclein, and spread through neural circuits. **(F)** Therapeutic strategies. Potential approaches include stabilizing physiological conformations, preventing oligomerization, modulating membrane interactions, restoring mitochondrial quality control, and blocking propagation. GWAS, genome-wide association studies; KO, knockout; NAC, non-amyloid-β component; OXPHOS, oxidative phosphorylation; PD, Parkinson’s disease; SNARE, soluble N-ethylmaleimide-sensitive factor attachment protein receptor; SNCA, gene encoding α-synuclein; TRAP1, tumor necrosis factor receptor-associated protein 1; ↑, increased; ↓, decreased.

These advances have reshaped our understanding of α-synuclein and, by extension, PD. α-Synuclein is not merely an aggregating presynaptic protein but also a regulator of neuronal homeostasis whose dysfunction disrupts vesicle trafficking, lipid composition, mitochondrial energetics, and intercellular communication (Treviño et al., 2025). Pathological progression appears to be driven by shifts in its structural equilibrium toward toxic oligomers and by prion-like propagation across neural systems (Figure 4).

This integrated framework compels a shift in diagnostic and therapeutic strategy: biomarkers must capture early, dynamic changes in α-synuclein conformation and organellar stress, and treatments must stabilize physiological multimers, prevent toxic oligomer formation, modulate membrane interactions, and interrupt trans-synaptic spread. For clinicians and neuroscientists, α-synuclein represents both a mechanistic cornerstone and a prime therapeutic target in efforts to delay or prevent the onset of irreversible neuronal loss.

### 3.8 GBA1: lysosomal dysfunction and mitochondria–lysosome crosstalk

Variants in GBA1, which encodes the lysosomal enzyme glucocerebrosidase, are among the most important genetic risk factors for Parkinson’s disease. Biallelic pathogenic variants cause Gaucher disease, whereas heterozygous variants increase susceptibility to PD, placing GBA1 at the interface between monogenic lysosomal disease and complex neurodegeneration (Volta, 2023).

Glucocerebrosidase participates in lysosomal sphingolipid degradation, and reduced enzyme activity disrupts lysosomal homeostasis, autophagic processing, and α-synuclein clearance. These alterations can establish a bidirectional pathogenic relationship in which glucocerebrosidase deficiency promotes α-synuclein accumulation, while α-synuclein-related proteostatic stress further compromises lysosomal function (Volta, 2023).

GBA1 dysfunction also perturbs direct communication between mitochondria and lysosomes. In dopaminergic neurons derived from patients carrying GBA1 variants, mitochondria–lysosome contacts were abnormally prolonged because of defective regulation of the Rab7 GTPase-activating protein TBC1D15. Increasing glucocerebrosidase activity restored normal contact dynamics, indicating that lysosomal enzyme deficiency can directly alter mitochondrial network regulation (Kim et al., 2021). These findings position GBA1 as a paradigmatic mitochondrial–lysosomal bridge in PD, in which lysosomal dysfunction, defective organelle communication, impaired degradative pathways, and α-synuclein accumulation reinforce one another.

### 3.9 VPS35 and retromer-dependent control of mitochondrial dynamics

VPS35 encodes a core component of the retromer complex, which regulates endosomal cargo sorting and recycling. The pathogenic VPS35 D620N variant causes autosomal-dominant PD and links vesicular trafficking defects to altered mitochondrial dynamics and bioenergetics (Lucchesi et al., 2025).

The D620N variant promotes excessive mitochondrial fission and fragmentation by increasing the interaction of VPS35 with dynamin-like protein 1, also known as DLP1 or DRP1. This interaction accelerates the trafficking of mitochondrial DLP1 complexes through mitochondrial-derived vesicles to lysosomes for degradation, disrupting the normal balance between mitochondrial fusion and fission (Wang et al., 2016). Fibroblasts carrying pathogenic VPS35 variants also exhibit impaired complex I and II activity and reduced bioenergetic efficiency (Lucchesi et al., 2025).

VPS35 therefore provides a mechanistic connection between retromer-dependent vesicular trafficking, lysosomal degradation, and mitochondrial network maintenance. Although it does not directly mediate mitochondrial surveillance in the same manner as PINK1 and Parkin, VPS35 dysfunction converges on mitochondrial fragmentation, impaired organelle quality control, and reduced neuronal stress tolerance.

### 3.10 CHCHD2, CHCHD10, and mitochondrial cristae integrity

CHCHD2 encodes a coiled-coil-helix-coiled-coil-helix domain protein localized primarily in the mitochondrial intermembrane space. Rare CHCHD2 missense variants have been associated with autosomal-dominant, generally late-onset PD. CHCHD2 participates in oxidative phosphorylation, mitochondrial apoptosis, cristae maintenance, neuronal function, and cellular responses to metabolic and oxidative stress (Lucchesi et al., 2025).

Loss or pathogenic alteration of CHCHD2 affects mitochondrial morphology and biogenesis and has been associated with reduced respiratory-chain activity, particularly involving complexes I and IV. Experimental loss of CHCHD2 disrupts cristae architecture, destabilizes cytochrome c, increases sensitivity to oxidative stress, and promotes age-dependent dopaminergic neuronal degeneration and motor impairment (Meng et al., 2017; Lucchesi et al., 2025). CHCHD2 also interacts functionally with PINK1 and other mitochondrial quality-control proteins, linking cristae and respiratory-chain abnormalities with impaired mitochondrial stress responses (Lucchesi et al., 2025).

CHCHD10 is a homologous mitochondrial intermembrane-space protein that can form a functional complex with CHCHD2. PD-associated CHCHD2 mutations can impair CHCHD10 stability and disrupt the CHCHD2–CHCHD10 complex, providing additional evidence that dysfunction of this protein family compromises cristae organization and mitochondrial integrity (Zhou et al., 2019). However, CHCHD10 variants are more firmly associated with amyotrophic lateral sclerosis, frontotemporal dementia, and related neurodegenerative syndromes than with classical PD, and its role in PD should therefore be interpreted cautiously.

### 3.11 FBXO7 and parkin-dependent mitochondrial quality control

Mutations in FBXO7 cause a rare autosomal-recessive juvenile or early-onset parkinsonian–pyramidal syndrome and connect ubiquitin-dependent proteostasis with mitochondrial quality control. FBXO7 encodes an F-box adaptor protein involved in SKP1–Cullin–F-box ubiquitin E3-ligase activity and substrate targeting for proteasomal degradation (Lucchesi et al., 2025).

FBXO7 also interacts with Parkin and promotes its recruitment to damaged mitochondria, thereby facilitating mitophagy. FBXO7 deficiency in dopaminergic cells and patient-derived fibroblasts is associated with reduced mitochondrial membrane potential, impaired complex I activity, decreased NAD^+^ and ATP levels, and increased reactive oxygen species. Excessive oxidative stress can further deplete NAD^+^ and ATP through poly (ADP-ribose) polymerase activation, amplifying energetic failure (Lucchesi et al., 2025).

Although FBXO7-associated parkinsonism is uncommon, its interaction with Parkin reinforces the broader principle that ubiquitin-dependent proteostasis and mitochondrial clearance are functionally integrated in PD.

### 3.12 LRRK2: reconciling its dual kinase and GTPase functions in mitochondrial regulation

Mutations in *LRRK2* (leucine-rich repeat kinase 2) are the most common cause of autosomal-dominant PD and a major genetic risk factor in sporadic cases (Rocha et al., 2022). Large genome-wide association studies continue to reinforce the central role of *LRRK2* variation in PD susceptibility (Lai et al., 2021). The gene encodes a large, multidomain protein of 2,527 amino acids, characteristic of the ROCO family, containing an ROC GTPase domain, a COR (C-terminal of ROC) dimerization module, and a serine/threonine kinase domain similar to receptor-interacting and mixed-lineage kinases (Deniston et al., 2020). Additional N- and C-terminal domains—including ankyrin repeats, leucine-rich repeats, and a WD40 domain—provide extensive scaffolding potential, positioning LRRK2 as an integrator of diverse intracellular signaling pathways (Myasnikov et al., 2021). Table 7 summarizes the role of LRRK2 as a kinase–GTPase regulator intersecting mitochondrial, lysosomal, and immune pathways in Parkinson’s disease.

**TABLE 7:** LRRK2 — kinase–GTPase regulator intersecting mitochondrial and lysosomal homeostasis.

Molecular architecture	Mechanistic events (→)	Mitochondrial and cellular consequences	Evidence and clinical relevance
ROC GTPase + COR dimerization + kinase domain + LRR/ANK/WD40 scaffolds	ROC–COR dimer dynamics → regulate kinase activation; kinase → phosphorylates ROC domain → modulates GTPase cycling	Alters Rab GTPase phosphorylation; disrupts endolysosomal sorting; perturbs cytoskeleton; modifies mitochondrial dynamics	Cryo-EM structures; pathogenic G2019S/R1441 mutations → ↑ kinase or ↓ GTPase activity
Rab GTPase phosphorylation network	RAB10/12/29, etc., → phosphorylated → altered vesicle trafficking, lysosomal function	Lysosomal stress; impaired autophagy–endosomal flux; metabolic alterations	Human PD tissue proteomics; iPSC-derived neurons
Mitochondrial effects	Mutants → ↑ Drp1 recruitment → fragmentation → ↓ ATP → altered network connectivity	Context-dependent mitochondrial vulnerability; possibly secondary to lysosomal dysfunction	Patient fibroblasts; neuronal cultures
Immunoregulation	LRRK2 ↑ in microglia/macrophages; modulates NFAT translocation; pathogen handling	Links PD genetics to peripheral + central inflammation	GWAS links to IBD, leprosy, and immune activation phenotypes
Therapeutic implications	CNS-penetrant kinase inhibitors; need to preserve physiological GTPase cycling; biomarkers of Rab phosphorylation and lysosomal stress	One of the most advanced genetically targeted PD therapeutic pipelines	Early clinical trials show pathway suppression

ANK, ankyrin repeat domain; ATP, adenosine triphosphate; CNS, central nervous system; COR, C-terminal of ROC, domain; Cryo-EM, cryogenic electron microscopy; Drp1, dynamin-related protein 1; GDP, guanosine diphosphate; GTP, guanosine triphosphate; GWAS, genome-wide association studies; IBD, inflammatory bowel disease; iPSC, induced pluripotent stem cell; LRR, leucine-rich repeat domain; LRRK2, leucine-rich repeat kinase 2; NFAT, nuclear factor of activated T cells; PD, Parkinson’s disease; Rab, Ras-related protein Rab; RAB10/12/29, Ras-related protein Rab 10/12/29; ROC, Ras of complex proteins GTPase domain; WD40, WD40-repeat domain; ↑, increased or enhanced; ↓, decreased or reduced; →, leads to, promotes, or contributes to; +, together with or combined with;/, and/or.

The mechanistic architecture of LRRK2 has long raised the question of whether it functions primarily as a GTPase-regulated kinase or a kinase-regulated GTPase (Taymans et al., 2023). A consensus has emerged that both enzymatic activities are tightly coupled and reciprocally modulate each other. Dimerization of LRRK2 is essential for full catalytic activity, influencing both GTP binding and kinase function (Taymans et al., 2023). Early structural and biochemical studies suggested that pathogenic mutations, such as G2019S (kinase domain) and R1441 C/G/H (ROC domain), shift this equilibrium by either enhancing kinase activity or reducing GTP hydrolysis (Xenias et al., 2022). More recent analyses using cryogenic electron microscopy and real-time phosphorylation assays show that ROC–COR dimer dynamics allosterically regulate kinase activation, while the kinase domain phosphorylates sites within the ROC domain that modulate GTPase cycling (Watanabe et al., 2020; Myasnikov et al., 2021). This bidirectional regulatory logic is further supported by the identification of endogenous interactors, such as ArfGAP1, a GTPase-activating protein that stimulates LRRK2 GTP hydrolysis and is phosphorylated by LRRK2, highlighting a regulatory loop central to its physiological function (Lewis et al., 2007).

Clinically, pathogenic *LRRK2* mutations produce a phenotype that frequently resembles sporadic PD, although Lewy pathology and clinical manifestations remain variable (Grover et al., 2022). Increased LRRK2 kinase activity and phosphorylation of Rab substrates have therefore been pursued as tractable therapeutic targets (Lee et al., 2010). Brain-penetrant small-molecule inhibitors have included DNL201 and DNL151, subsequently designated BIIB122. Early clinical studies demonstrated inhibition of LRRK2 pathway biomarkers, including reductions in phosphorylated LRRK2 and Rab10, supporting pharmacological target engagement.

*LRRK2* inhibition has nevertheless raised safety and efficacy concerns. Chronic inhibition in non-human primates produced reversible accumulation of lamellar bodies within type II pneumocytes, a histopathological change consistent with the physiological role of LRRK2 in lysosomal processing in the lung (Fuji et al., 2015; Baptista et al., 2020). These alterations were not accompanied by clear impairment of pulmonary function in the reported studies, but they prompted continued respiratory surveillance in clinical development. In addition, the Phase 2b LUMA trial of BIIB122 in early idiopathic PD did not meet its primary or secondary efficacy endpoints, and development for idiopathic PD was discontinued (Sammler et al., 2026). Evaluation in genetically defined carriers of pathogenic LRRK2 variants continues, where stronger pathway dependence and biomarker-guided selection may provide a more appropriate test of the therapeutic hypothesis. Thus, LRRK2 remains a biologically compelling and pharmacologically tractable target, but clinical disease modification has not yet been demonstrated (Lang et al., 2026).

The cellular reach of LRRK2 is extensive. Studies of gain- and loss-of-function in diverse models—from primary neurons to human pluripotent stem cell–derived dopaminergic cultures—demonstrate that LRRK2 influences cytoskeletal dynamics, neurite elongation, vesicular trafficking, endolysosomal sorting, autophagy, and innate immune signaling (Alessi and Sammler, 2018; Wallings et al., 2024). A unifying theme emerging from recent work is the central role of LRRK2 in Rab GTPase phosphorylation, with RAB10, RAB12, RAB29, and others serving as highly regulated substrates that control organellar trafficking and vesicle dynamics (Pfeffer, 2023). Perturbations in this Rab phosphorylation network are now thought to link LRRK2 dysfunction to impaired lysosomal homeostasis, one of the most reproducible pathogenic pathways in human PD tissue.

Animal models have corroborated the role of mutant LRRK2 in late-onset dopaminergic neurodegeneration, albeit incompletely (Seegobin et al., 2020). *Drosophila* expressing G2019S LRRK2 display progressive dopaminergic neuron loss and locomotor impairment, both of which can be ameliorated by coexpression of parkin, suggesting functional crosstalk between LRRK2 and mitochondrial quality-control pathways (Hindle et al., 2013). In mammalian systems, knock-in mice expressing pathogenic variants develop subtle but progressive synaptic and mitochondrial defects, with aging exacerbating underlying vulnerabilities (Volta, 2023). Despite these advances, key questions remain regarding which LRRK2-dependent pathways are causally implicated in human disease and which represent compensatory cellular responses.

An important conceptual development has been the recognition of LRRK2 as an immunoregulatory protein (Wallings and Tansey, 2019). Strong associations between *LRRK2* variants and inflammatory bowel disease, leprosy, and tuberculosis highlight its broad role in host defense (Whiffin et al., 2020; Wallings et al., 2024). LRRK2 is highly expressed in macrophages, microglia, and B lymphocytes, where it modulates inflammatory responses by regulating the nuclear translocation of NFAT transcription factors and influencing lysosomal dynamics during pathogen handling (Russo et al., 2022). These findings have renewed interest in PD as a disorder with a significant immunometabolic component, raising the possibility that systemic inflammation may amplify neurodegenerative cascades in genetically susceptible individuals.

The impact of LRRK2 on mitochondrial biology remains an area of active investigation (Singh et al., 2019). A subset of LRRK2 localizes to mitochondria, and pathogenic variants induce abnormalities in mitochondrial morphology, membrane potential, and bioenergetics. Mitochondrial fragmentation associated with G2019S expression has been linked to increased recruitment of the dynamin-related protein Drp1, and modulation of mitochondrial fission–fusion dynamics can mitigate LRRK2-induced toxicity in neuronal cultures (Singh et al., 2019). Human fibroblasts from carriers of the G2019S mutation exhibit decreased ATP production and altered mitochondrial connectivity, phenotypes that align with clinical observations of subtle metabolic deficits in *LRRK2*-associated PD (Delcambre et al., 2020). However, it remains uncertain whether mitochondrial abnormalities are primary contributors to degeneration or secondary effects of disrupted lysosomal and trafficking pathways.

**FIGURE 5:**
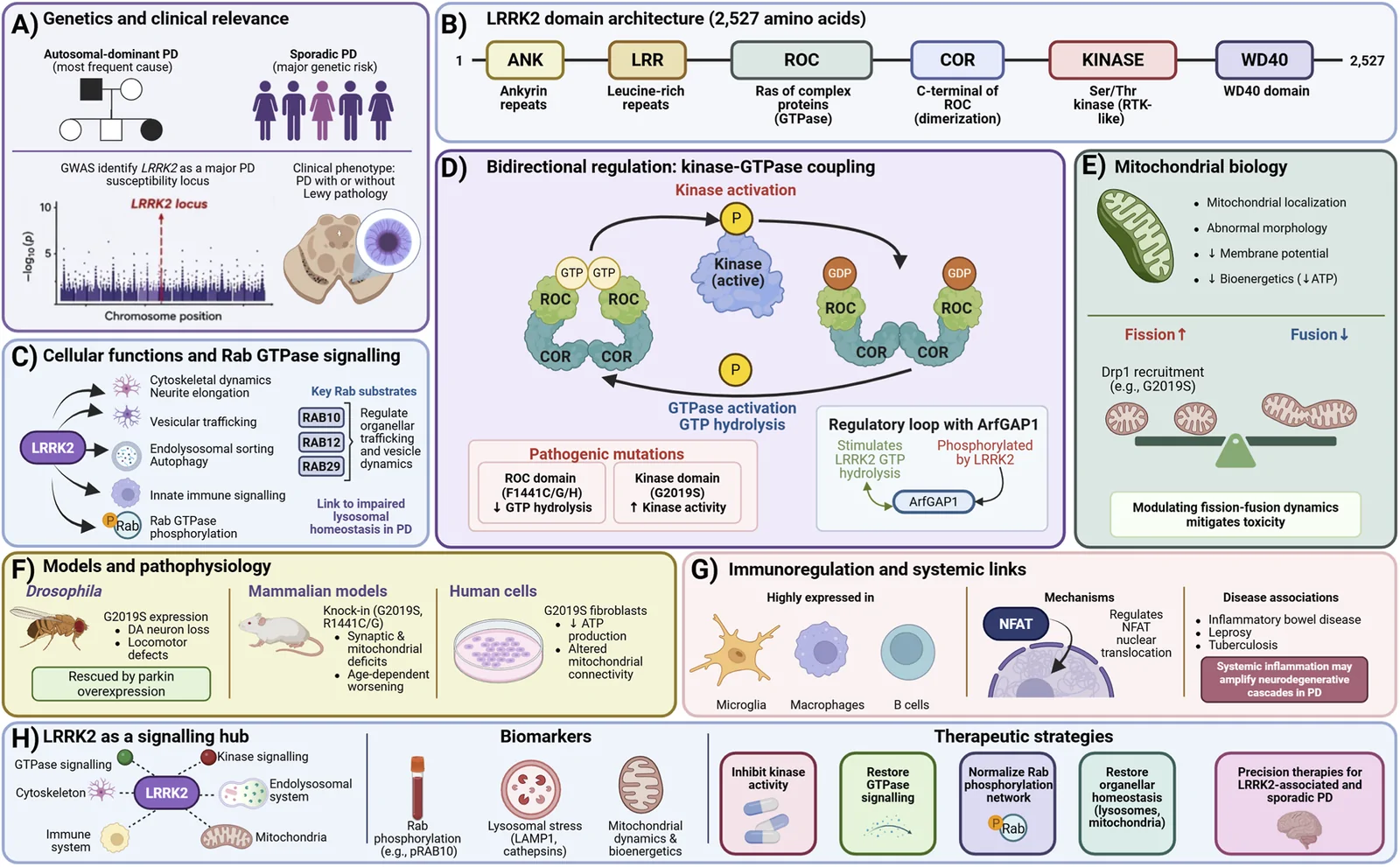
LRRK2 as a kinase–GTPase signaling hub linking vesicular trafficking, mitochondrial dysfunction, and immune responses in Parkinson’s disease. LRRK2 integrates enzymatic signaling, organellar homeostasis, and inflammatory responses relevant to Parkinson’s disease. **(A)** Genetic and clinical relevance. Pathogenic LRRK2 variants are a major cause of autosomal-dominant Parkinson’s disease, and genome-wide association studies identify LRRK2 as an important susceptibility locus in sporadic disease. Clinical phenotypes may occur with or without Lewy body pathology. **(B)** Domain architecture. LRRK2 is a multidomain ROCO-family protein containing ankyrin repeats, leucine-rich repeats, ROC GTPase, COR dimerization, kinase, and WD40 domains. **(C)** Cellular functions and Rab signaling. LRRK2 regulates cytoskeletal dynamics, neurite elongation, vesicular trafficking, endolysosomal sorting, autophagy, and innate immune signaling, partly through phosphorylation of Rab GTPases such as Rab10, Rab12, and Rab29. **(D)** Kinase–GTPase coupling. ROC–COR dimerization, GTP hydrolysis, kinase activation, and ArfGAP1-dependent regulation form a bidirectional enzymatic loop disrupted by pathogenic mutations. **(E–G)** Pathophysiological effects. LRRK2 dysfunction alters mitochondrial morphology, membrane potential, bioenergetics, fission–fusion balance, experimental disease phenotypes, and immune-cell responses. **(H)** Translational implications. LRRK2 acts as a systems-level signaling hub, supporting biomarkers based on Rab phosphorylation, lysosomal stress, and mitochondrial dynamics, and therapeutic strategies aimed at kinase inhibition, restoration of GTPase signaling, Rab normalization, and organellar homeostasis. ANK, ankyrin repeats; ATP, adenosine triphosphate; COR, C-terminal of ROC; Drp1, dynamin-related protein 1; GDP, guanosine diphosphate; GTP, guanosine triphosphate; GWAS, genome-wide association studies; LAMP1, lysosomal-associated membrane protein 1; LRR, leucine-rich repeats; LRRK2, leucine-rich repeat kinase 2; NFAT, nuclear factor of activated T cells; PD, Parkinson’s disease; Rab, Ras-related protein Rab; ROC, Ras of complex proteins; WD40, WD40-repeat domain; ↑, increased; ↓, decreased.

Overall, emerging data suggests that LRRK2 functions as a hub coordinating GTPase and kinase signaling across endolysosomal, cytoskeletal, mitochondrial, and immune systems. This systems-level perspective requires recalibrating diagnostic and therapeutic strategies: biomarkers must capture LRRK2-driven alterations in Rab phosphorylation, lysosomal stress, and mitochondrial dynamics, while therapies must be tailored not only to kinase inhibition but also to restoring physiological GTPase signaling and organellar homeostasis (Figure 5). For clinicians and neuroscientists, LRRK2 exemplifies the paradigm shift in PD research, moving from a dopamine-centric framework toward a mechanistic understanding rooted in intracellular trafficking, metabolic resilience, and neuroimmune regulation.

## 4 Interconnected mitochondrial pathways in Parkinson’s disease pathogenesis

### 4.1 Mitochondrial–lysosomal crosstalk, vesicle trafficking, and autophagic flux

Current findings show that mitochondria and lysosomes form dynamic physical and functional contacts that coordinate organelle dynamics, calcium signaling, lipid exchange, metabolic adaptation, and mitochondrial turnover in neurons. In PD, this bidirectional communication is disrupted by α-synuclein accumulation (Giamogante et al., 2024) and by lysosomal risk genes, most prominently GBA1 (Kim et al., 2021). Reduced glucocerebrosidase activity alters lysosomal sphingolipid composition, impairs proteolytic and autophagic flux, and promotes α-synuclein accumulation. These changes secondarily compromise mitochondrial respiration, membrane potential, morphology, and mitophagic clearance. In turn, mitochondrial oxidative and proteostatic stress can further impair lysosomal function, establishing a self-amplifying cycle of organelle dysfunction. VPS35-dependent retromer defects and LRRK2-mediated Rab dysregulation provide additional routes through which vesicular trafficking abnormalities disrupt mitochondrial–lysosomal coordination. When lysosomal function is compromised, cells secrete mitochondria within extracellular vesicles as an alternative quality-control mechanism, bypassing canonical degradation pathways (Liang et al., 2023).

Impaired mitochondria-initiated crosstalk with lysosomes, mediated by factors such as LManVI, further downregulates PGC-1α and exacerbates mitochondrial gene expression defects in a positive feedback loop (Li S. et al., 2025). Vesicle trafficking, including mitochondrial-derived vesicles, integrates with lysosomal delivery systems to clear oxidized cargo, but PD-associated defects in Rab7 and endosomal pathways disrupt this coordination (Kiraly et al., 2025). These mechanisms function independently of selective mitophagy and highlight a broader network of organelle crosstalk that maintains neuronal proteostasis under chronic stress.

### 4.2 Intercellular mitochondrial transfer as a compensatory mechanism in neuronal–glial networks

Glial cells transfer functional mitochondria to stressed neurons via tunneling nanotubes and extracellular vesicles, providing metabolic support and enhancing neuronal resilience. In PD models, microglia donate healthy mitochondria to dopaminergic neurons burdened with α-synuclein, while astrocytes release mitochondria-derived extracellular vesicles that restore energy metabolism. This transfer, facilitated by myosin-10, is bidirectional; neurons can also export damaged components to glia for detoxification (Xu et al., 2026).

Recent *in vivo* evidence shows that satellite glial cells in dorsal root ganglia form nanotube connections to transfer mitochondria, protecting sensory neurons from chronic injury, a mechanism proposed to extend to central dopaminergic circuits (Liang et al., 2025). This intercellular exchange serves as a non-cell-autonomous compensatory pathway distinct from intracellular quality control and may limit bioenergetic failure during early PD pathogenesis.

### 4.3 Mitochondrial contributions to neuroinflammation via damage-associated molecular patterns

Mitochondrial damage releases damage-associated molecular patterns (mtDAMPs), including mtDNA, cytochrome c, cardiolipin, ATP, and formyl peptides, which activate pattern-recognition receptors on microglia and astrocytes (Brooks et al., 2026). In PD, these molecules amplify innate immune responses, promoting pro-inflammatory cytokine release and oxidative stress that exacerbate neuronal loss.

MtDAMPs are packaged into extracellular vesicles, enabling paracrine signaling that spreads neuroinflammation beyond the initial site of mitochondrial impairment (Lin et al., 2022). This pathway links mitochondrial dysfunction directly to sustained glial activation and is observed in both sporadic and genetic PD subtypes, independent of Lewy body formation or classical mitophagy defects.

## 5 Reframing foundational assumptions and persistent uncertainties in Parkinson’s disease biology

### 5.1 The energetic–electrophysiological paradox: Why do SNc dopaminergic neurons fail first?

A central challenge in contemporary neurodegeneration research is explaining why dopaminergic neurons of the substantia nigra pars compacta (SNc) are so selectively vulnerable, despite the widespread expression of PD-related genes. These neurons occupy a uniquely precarious biochemical niche.

A leading structural explanation for this selective vulnerability is the exceptional size and complexity of their axonal arbor. Individual nigrostriatal neurons form extremely extensive, predominantly unmyelinated axonal trees containing very large numbers of striatal release sites. Using a quantitative energetic model, Pissadaki and Bolam proposed that action-potential propagation throughout this arbor, followed by restoration of transmembrane ion gradients, imposes a disproportionately high and continuous ATP demand (Pissadaki and Bolam, 2013). This burden is compounded by the energetic requirements of axonal transport, synaptic vesicle cycling, dopamine synthesis and packaging, and maintenance of distant terminals. Consequently, even modest impairment of mitochondrial ATP production or distribution may cause energetic demand to exceed supply across the axonal network, making arbor size itself a structural determinant of reduced mitochondrial resilience.

Autonomous pacemaking provides an additional source of sustained metabolic stress. Adult SNc dopaminergic neurons use L-type calcium channels, including CaV1.3-containing channels, during pacemaking, producing recurrent cytosolic calcium elevations that must be buffered by the endoplasmic reticulum and mitochondria. Mitochondrial calcium uptake can stimulate oxidative phosphorylation to match energetic demand, but it may also increase respiratory-chain activity and ROS production, particularly when respiratory reserve is limited, generating a metabolic profile resembling “physiological overclocking” (Guzman et al., 2010; Surmeier et al., 2017; Santana-Roman et al., 2025). This mechanism provided the rationale for testing dihydropyridine calcium-channel blockers as potential disease-modifying therapies. However, in the Phase three STEADY-PD III trial, immediate-release isradipine did not slow clinical progression over 36 months in patients with early PD (The Parkinson Study Group STEADY-PD III Investigators, 2020). Thus, calcium-dependent pacemaking remains a plausible contributor to selective neuronal vulnerability, but systemic pharmacological blockade with isradipine has not demonstrated clinical neuroprotection. The negative trial may reflect insufficient target engagement, dose limitations, channel selectivity, timing of intervention, or the multifactorial nature of neuronal vulnerability; nevertheless, it argues against presenting CaV1.3 inhibition as an established disease-modifying strategy.

Intracellular dopamine dynamics further amplify this vulnerability. Cytosolic dopamine is inherently prone to oxidation, forming quinones and hydrogen peroxide during both enzymatic and non-enzymatic metabolism. Any reduction in vesicular monoamine transporter 2 (VMAT2) activity—whether due to impaired ATP supply, altered trafficking, or proteostatic stress—increases cytosolic dopamine levels and accelerates oxidative injury (Lohr et al., 2014). Additionally, SNc neurons contain high iron concentrations and comparatively depleted antioxidant reserves, creating a pro-oxidant microenvironment that magnifies metabolic insults (Ndayisaba et al., 2019).

These intrinsic risk factors interact with network-level stressors. Dense glutamatergic input from the subthalamic nucleus predisposes SNc neurons to excitotoxicity when ATP-dependent ion homeostasis fails (Smith and Grace, 1992; Rodriguez et al., 1998; Santana-Roman et al., 2025). Mitochondrial or endoplasmic reticulum stress disrupts calcium buffering, triggers mitochondrial calcium overload, reduces ATP generation, and activates cell death pathways (Zhao and Sheng, 2025). No single mechanism fully accounts for selective vulnerability; instead, converging factors—including neuromelanin accumulation, impaired proteostasis, microglial priming, and age-related mitochondrial decline—appear to erode neuronal resilience over decades (Surmeier et al., 2017; Santana-Roman et al., 2025). This convergence model requires a shift from linear causality to systems-level explanations of neurodegeneration.

**FIGURE 6:**
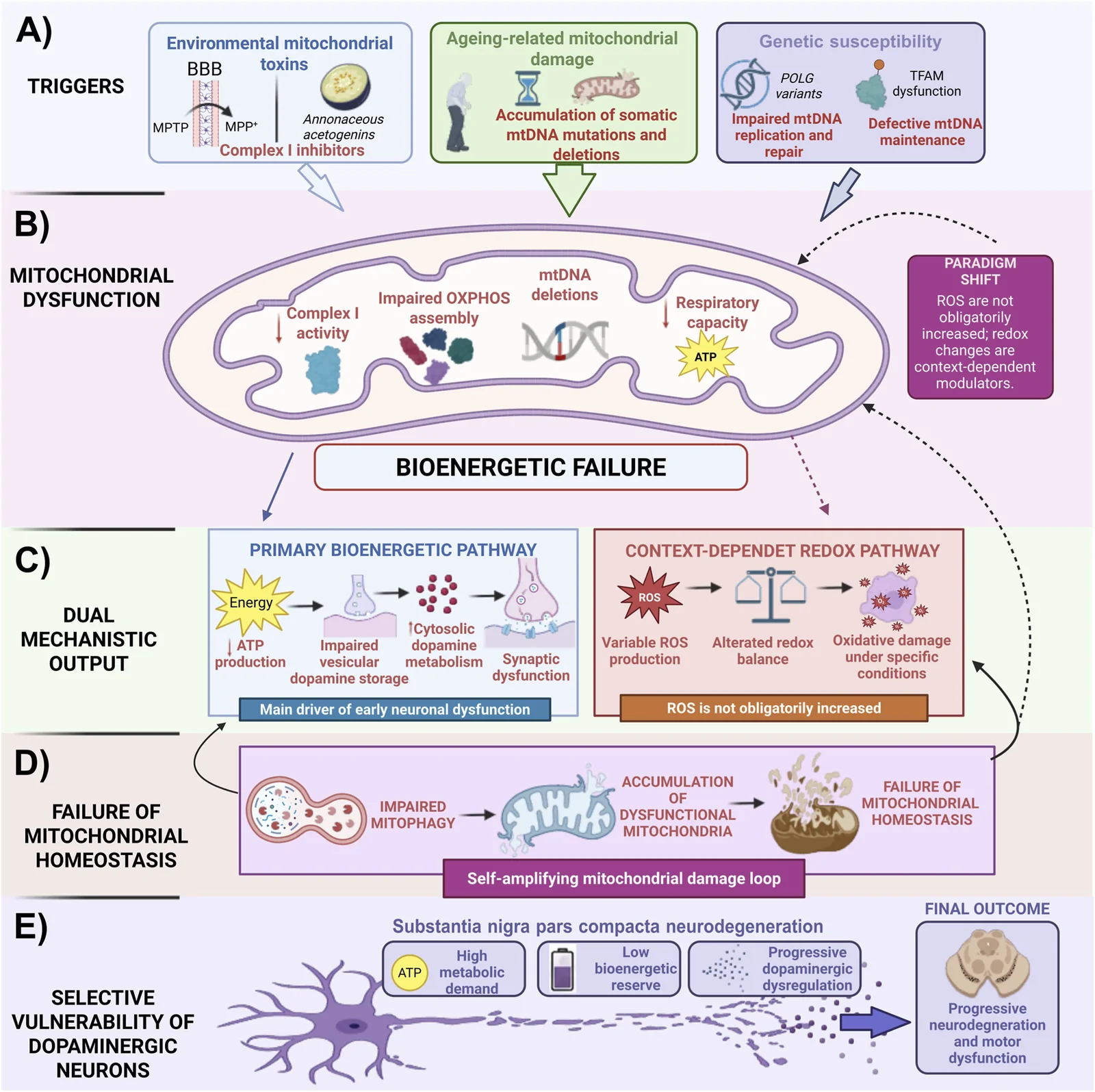
Systems-level determinants of selective vulnerability of substantia nigra pars compacta dopaminergic neurons in Parkinson’s disease. Substantia nigra pars compacta dopaminergic neurons are selectively vulnerable because multiple intrinsic, mitochondrial, network-level, and age-related stressors converge over time. **(A)** Energetic–electrophysiological paradox. The vast, largely unmyelinated axonal arbor and numerous release sites of SNc dopaminergic neurons impose a high basal ATP demand, while autonomous pacemaking and calcium entry further increase metabolic load and mitochondrial reactive oxygen species production. Dopamine metabolism, impaired vesicular storage, high iron content, and limited antioxidant defenses simultaneously create a pro-oxidant intracellular environment. Although calcium-dependent vulnerability remains mechanistically plausible, isradipine did not slow disease progression in the Phase 3 STEADY-PD III trial. **(B)** Mitochondrial dysfunction. Complex I impairment may reduce NADH oxidation, electron flow, and ATP production, while increasing susceptibility to redox imbalance. However, mitochondrial dysfunction may act either as an initiating insult or as an amplifier of pre-existing cellular stress. **(C)** Network vulnerability. Excitotoxic input from the subthalamic nucleus, neuroinflammation, and age-related mitochondrial decline further reduce neuronal resilience, supporting a cumulative-stress model rather than a single-hit mechanism. **(D)** Lessons from model systems. Divergent findings across *Drosophila*, mammalian models, and human cells indicate that mitochondrial mechanisms depend on species, developmental stage, and cellular context. **(E)** Unresolved questions. Key challenges include defining thresholds of irreversible mitochondrial failure, identifying early actionable events, and clarifying how genetic, environmental, and aging-related factors converge during disease progression. ATP, adenosine triphosphate; Ca^2+^, calcium ion; CI, complex I; ER, endoplasmic reticulum; Fe, iron; GDP, guanosine diphosphate; GSH, glutathione; H_2_O_2_, hydrogen peroxide; NADH, reduced nicotinamide adenine dinucleotide; PD, Parkinson’s disease; PINK1, PTEN-induced kinase 1; ROS, reactive oxygen species; SNc, substantia nigra pars compacta; SOD, superoxide dismutase; STN, subthalamic nucleus; VMAT2, vesicular monoamine transporter 2; ↑, increased; ↓, decreased.

The major conceptual uncertainties surrounding selective dopaminergic vulnerability, mitochondrial dysfunction, model-system discrepancies, and unresolved translational questions are summarized in Figure 6.

### 5.2 Complex I: Pathogenic trigger, downstream amplifier, or both?

Complex I dysfunction has long been considered a mechanistic cornerstone of PD, based on observations that mitochondrial toxins such as MPTP, rotenone, and annonacin induce parkinsonism in humans and animal models. However, it remains uncertain whether inhibition of complex I is a primary pathogenic event or a secondary amplifier of cellular stress. The specificity of these agents is limited—rotenone, for example, destabilizes microtubules independently of respiratory inhibition—so their pathogenicity may reflect broader metabolic disruption (Passmore et al., 2017).

Nonetheless, experimental evidence continues to link impaired NADH dehydrogenase activity with dopaminergic vulnerability (Marella et al., 2009). Introduction of the yeast enzyme Ndi1p, which is resistant to MPTP and rotenone, protects dopaminergic neurons in mammalian systems, highlighting a causal relationship between respiratory insufficiency and neurodegeneration (Li et al., 2024). Genetic loss of Ndufs4, a complex I assembly factor, initially appeared to contradict this model because the SNc remained relatively intact (Quintana et al., 2010). However, subsequent work showed that residual complex I function persists via supercomplex stabilization, explaining partial regional sparing (Davoudi et al., 2014).

The most compelling evidence for complex I involvement comes from PINK1-associated disease (Morais et al., 2014). Complex I activity is consistently reduced in PINK1-deficient models and restoring electron transport through Ndi1p expression or small-molecule electron carriers, such as vitamin K2 rescues multiple phenotypes in flies (Vos et al., 2021). Whether these mechanisms generalize to mammalian neurons remains unresolved, but current data increasingly support a model in which modest, chronic complex I impairment contributes to PD pathogenesis through cumulative metabolic stress rather than acute bioenergetic collapse.

### 5.3 Reconciling discrepancies across species, experimental scales, and model systems

*Drosophila* genetics has been instrumental in establishing the relationship between PINK1, parkin, and mitochondrial maintenance (Park et al., 2025). Loss of either gene produces a convergent phenotype characterized by muscle degeneration, male sterility, and dopaminergic neuron loss, findings that have shaped the prevailing view of PINK1–parkin function (Yun et al., 2014). However, translating these discoveries to mammalian systems has revealed critical discrepancies that challenge overly linear interpretations of mitochondrial biology.

In flies, promoting mitochondrial fission rescues PINK1 or parkin deficiencies, whereas in mammalian neurons, the opposite occurs: enhancing mitochondrial fusion ameliorates deficits caused by the acute loss of either protein (Yang et al., 2008). Similarly, mitochondrial transport phenotypes diverge across species. PINK1 deficiency increases Miro abundance in *Drosophila* but reduces it in human cells, reflecting differences in turnover mechanisms and compensatory pathways (Bayne and Trempe, 2019).

These discrepancies underscore a conceptual shift: PD gene function is not defined by a single canonical pathway but reflects a network of context-dependent interactions that vary across species, developmental stages, and metabolic states (Santana-Roman et al., 2025). Mammalian neurons possess complex buffering mechanisms, including redundant mitophagy pathways, extensive chaperone networks, and age-dependent compensatory programs, that are absent or attenuated in invertebrates. This complexity explains both the muted phenotypes of many genetic mouse models and the late-onset nature of human disease, in which decades of accumulated microstress ultimately overwhelm compensatory reserves.

### 5.4 Mitophagy: Canonical pathway, context-dependent response, or experimental variable?

The discovery of the PINK1–parkin mitophagy pathway transformed conceptual frameworks for mitochondrial quality control (Waters et al., 2023). In cultured cells, mitochondrial depolarization triggers rapid PINK1 accumulation on the outer membrane, parkin recruitment, and ubiquitin-dependent elimination of damaged mitochondria (Pickrell and Youle, 2015). However, whether this pathway operates broadly and physiologically in human dopaminergic neurons remains highly contested.

Chemical uncouplers such as CCCP induce extreme, synchronous mitochondrial depolarization, conditions that do not reflect physiological mitochondrial stress (Hu D. et al., 2021; Hu N. et al., 2021). Moreover, tumor-derived cell lines and fibroblasts rely heavily on glycolysis and can eliminate mitochondria with minimal functional consequences, in sharp contrast to neurons, which depend on oxidative phosphorylation for continuous ATP generation. Evidence from mammalian neuronal models suggests that global mitophagy is rare under basal conditions and that alternative mechanisms—mitochondria-derived vesicles, local protein-level quality control, and receptor-mediated pruning—may predominate (Abeliovich and Gitler, 2016).

Notably, the observation that parkin rescues PINK1 mutant flies—despite being unable to promote mitophagy without PINK1—suggests that mitophagy is only one component of a broader mitochondrial maintenance network. *In vivo*, mitochondrial quality control likely depends on graded, modular processes rather than a single binary clearance pathway. The emerging conclusion is not that mitophagy is irrelevant to PD, but that its role is context-specific, cell-type-dependent, and unlikely to be the sole initiating defect in human disease.

## 6 Discussion

Over the past 2 decades, research on genes associated with PD has converged on a striking insight: despite the heterogeneity of clinical and genetic presentations, many PD-linked proteins intersect with pathways governing mitochondrial integrity, dynamics, and stress responses (Nalls et al., 2019; Kim et al., 2024; Arias-Carrion et al., 2026).

Studies of PINK1, Parkin, DJ-1, LRRK2, and α-synuclein increasingly position mitochondrial dysfunction not as an ancillary feature of neurodegeneration but as a core pathogenic process shared across sporadic and familial disease (Chung et al., 2016). This mechanistic convergence has stimulated interest in circulating and genetic indicators of mitochondrial dysfunction, including phosphorylated PINK1/Parkin-related measures, oxidized or 4-HNE-modified DJ-1 species, plasma irisin, and polygenic risk scores derived from oxidative-phosphorylation genes. Erythrocyte oxidized DJ-1 and 4-HNE-modified DJ-1 have been associated with disease stage and dopaminergic vulnerability, while plasma irisin has been reported to correlate inversely with α-synuclein burden and motor impairment (Chen et al., 2025; Usha Kiran et al., 2026). OXPHOS-based polygenic risk scores may also identify subgroups with greater inherited mitochondrial susceptibility. However, these candidates should be regarded as investigational rather than validated biomarkers for pre-motor detection. Most are supported by individual or limited-cohort studies, and their analytical reproducibility, biological specificity, stability across disease stages and treatments, and incremental value beyond established clinical, imaging, or α-synuclein-based markers remain uncertain. Independent replication, standardized assays, longitudinal studies, and prospective evaluation in prodromal and genetically at-risk populations will be required before they can support clinical diagnosis or patient stratification. Large-scale genome-wide association studies nevertheless reinforce the broader biological rationale by showing that risk variation in pathways containing *SNCA*, *LRRK2*, and mitochondrial-function genes contributes to susceptibility across familial and sporadic PD (Nalls et al., 2019; Kim et al., 2024; Arias-Carrion et al., 2026).

Therapeutic strategies intended to restore mitochondrial resilience are increasingly diverse and can be grouped according to the mitochondrial process they aim to modify. In primary mitochondrial disorders, established management includes avoidance of catabolic stressors and mitochondrial-toxic drugs, treatment of organ-specific complications, and selected use of cofactors or antioxidants such as CoQ10, riboflavin, thiamine, biotin, alpha-lipoic acid, N-acetylcysteine, and idebenone. Their clinical benefit is heterogeneous and is most clearly established in specific deficiency states or defined mitochondrial diseases, such as primary CoQ10 deficiency or Leber hereditary optic neuropathy, rather than in PD generally (Gruosso et al., 2021; Tinker et al., 2021; Ball et al., 2025).

Experience in PD demonstrates that mechanistic plausibility does not necessarily translate into disease modification. In the Phase three QE3 trial, high-dose CoQ10 was safe but did not slow functional decline in early PD, and the study was terminated for futility (The Parkinson Study Group QE3 Investigators, 2014). Similarly, the Phase three SURE-PD3 trial found that inosine-mediated elevation of serum and cerebrospinal-fluid urate did not reduce the rate of clinical progression and increased the risk of adverse effects such as nephrolithiasis (The Parkinson Study Group Sure-PD3 Investigators, 2021). More recently, once-weekly exenatide did not improve the primary clinical outcome or provide evidence of disease modification in the Phase three Exenatide-PD3 trial (Vijiaratnam et al., 2025). These negative trials do not exclude the involvement of oxidative, metabolic, or mitochondrial mechanisms in PD, but they show that correction of a single downstream pathway may be insufficient, particularly without demonstrated central target engagement, biological patient stratification, or intervention during prodromal disease.

Metabolic and lifestyle interventions, particularly supervised endurance or resistance exercise, may enhance mitochondrial biogenesis and respiratory capacity through PGC-1α-dependent pathways, while ketogenic or anaplerotic strategies may provide alternative energetic substrates in selected mitochondrial phenotypes, though their applicability to PD remains uncertain (Zong et al., 2024; Ball et al., 2025). In addition, strategies that promote adaptive metabolic rewiring may represent an emerging therapeutic direction. Enhancement of glycolysis through PGK1 activation has shown protective effects in experimental PD models and supportive associations in clinical databases, whereas hypoxia or hypoxia-like interventions have shown benefit in preclinical mitochondrial-disease and PD models by reducing oxygen-associated stress and activating HIF-1α-dependent programs. These approaches remain investigational and require careful assessment of safety, target engagement, and clinical translatability (Jain et al., 2016; Cai et al., 2019; Blume et al., 2025; Marutani et al., 2025). More targeted pharmacological approaches are being developed to stabilize mitochondrial membranes, modulate redox biology, improve NAD^+^ metabolism, stimulate mitochondrial biogenesis, enhance mitophagy, or restore electron transport, including compounds such as elamipretide, sonlicromanol/KH176, KL1333, nicotinamide riboside, omaveloxolone, vatiquinone/EPI-743, MitoQ, and PINK1/Parkin or USP30 modulators (Tinker et al., 2021; Hong et al., 2023; Zong et al., 2024; Ball et al., 2025). In parallel, gene-based approaches, mitochondrial replacement, mitochondrial transplantation, and strategies that promote intercellular mitochondrial transfer are emerging as more direct attempts to correct or bypass mitochondrial defects (Hong et al., 2023; Zong et al., 2024; Ball et al., 2025). In PD specifically, these interventions remain largely preclinical or early translational, but they reinforce the concept that mitochondrial dysfunction is not only a pathogenic mechanism but also a modifiable therapeutic target, particularly in biologically stratified patients with evidence of impaired mitochondrial quality control, bioenergetic failure, impaired metabolic flexibility, or neuroinflammatory mitochondrial signaling (Lucchesi et al., 2025).

Pharmacological activation of PINK1/Parkin signaling restores mitophagy and mitochondrial homeostasis. Small-molecule PINK1 activators, such as MTK458 and ABBV-1088, and USP30 inhibitors, such as MTX325, are in Phase 1 trials and have shown preclinical reduction of pathogenic α-synuclein and improved neuronal survival (Rosencrans et al., 2025; Hertz et al., 2024). BL-918 activates the PINK1/Parkin pathway by modulating mitochondrial membrane potential, ameliorating pathology in PD models (Wang et al., 2024). Mitochondrial transplantation and enhancement of intercellular transfer are emerging approaches, while PGC-1α upregulation through PARIS degradation supports mitochondrial biogenesis. These interventions target upstream resilience mechanisms and are advancing toward disease modification.

This convergence has shifted the field away from oxidative stress as the dominant explanatory framework toward a broader focus on bioenergetic failure, dysregulated mitochondrial quality control, impaired organellar cross-talk, and maladaptive stress signaling (Irrcher et al., 2010). Rather than a single linear cascade, PD increasingly appears to arise from cumulative disturbances in mitochondrial resilience, a dynamic process shaped by genetic vulnerability, aging, environmental exposures, and cell-type–specific metabolic demands.

To make this framework experimentally testable, mitochondrial resilience should not be inferred solely from the expression of individual quality-control proteins or from mitochondrial performance under basal conditions. Instead, it can be operationalized as the capacity of a neuronal mitochondrial network to withstand a defined stress, limit the resulting functional disruption, and restore bioenergetic and cellular homeostasis after the stress is removed. Conceptually, mitochondrial resilience could be quantified from three complementary dimensions: resistance, defined by the magnitude of functional loss during stress; recovery, defined by the rate and completeness of return toward baseline; and tolerance, defined by the stress dose or duration required to produce irreversible failure. This property can be assessed using standardized stress–recovery paradigms in which neurons are exposed to partial respiratory-chain inhibition, transient oxidative or calcium stress, or controlled mitochondrial depolarization, followed by longitudinal measurements during and after removal of the insult. Candidate readouts include spare respiratory capacity, the magnitude and reversibility of mitochondrial membrane-potential loss, ATP depletion and recovery kinetics, calcium recovery, mitophagic flux, and the stress intensity or duration required to induce irreversible depolarization or cell death. Under matched experimental conditions, a resilient neuron would exhibit smaller functional disruption, faster or more complete recovery, and a higher threshold for irreversible failure than a non-resilient neuron; a proposed resilience-enhancing mechanism should improve at least one prespecified recovery or failure-threshold endpoint without merely increasing basal mitochondrial activity.

No universally validated mitochondrial-resilience index currently exists, and fixed thresholds cannot yet be transferred across neuronal subtypes, stressors, or experimental platforms. Accordingly, recovery criteria, observation intervals, and normalization to basal energetic demand should be prospectively specified. A composite index integrating the depth of functional impairment, recovery time, recovery completeness, and cell survival may eventually permit comparisons across models, but such an index will require experimental and clinical validation.

Nevertheless, substantial uncertainties remain. A major challenge is distinguishing the direct mitochondrial effects of PD-related genes from secondary adaptations or systemic stress responses (Keane et al., 2011). Many studies demonstrating mitochondrial fragmentation, altered membrane potential, or impaired mitophagy have been conducted in overexpression systems or non-neuronal cell lines under experimental conditions that may exaggerate canonical pathways. The next phase of research must prioritize *in vivo* validation using physiological levels of gene expression, human induced pluripotent stem cell (iPSC)-derived dopaminergic neurons, and chronic stress paradigms that more accurately recapitulate the temporal trajectory of PD.

Another unmet need is a deeper understanding of how genetic and environmental exposures jointly shape mitochondrial vulnerability (Zanon et al., 2018). Epidemiological and mechanistic studies increasingly highlight the synergistic effects of pesticides, metabolic stressors, neuroinflammation, and age-dependent declines in proteostasis (Hollerhage, 2026). These interactions likely determine whether a neuron withstands or succumbs to cumulative mitochondrial insults. Precisely mapping these gene–environment interfaces will be central to predicting disease onset, progression, and therapeutic responsiveness.

The imperative now is translation. Restoring mitochondrial resilience offers a unified therapeutic strategy for PD, Alzheimer’s disease, Huntington’s disease, and amyotrophic lateral sclerosis, where shared defects in quality control, bioenergetics, and intercellular support increase neuronal vulnerability (Wadan et al., 2025). Clinical development of PINK1/Parkin enhancers and mtDAMP-targeted anti-inflammatory agents could shift treatment from symptomatic relief to early, mechanism-based neuroprotection. Precision approaches using OXPHOS-PRS or mtDAMP biomarkers may identify responders, enabling personalized interventions (Gouri et al., 2025). These advances highlight mitochondrial resilience as a modifiable hub with broad relevance across protein-misfolding neurodegenerative disorders.

If mitochondrial dysfunction constitutes an early, potentially reversible phase of PD, then therapeutic strategies must target mitochondrial resilience well before overt clinical symptoms emerge. This concept requires a new generation of biomarkers capable of detecting bioenergetic deficits, impaired mitochondrial turnover, or Rab/LRRK2 signaling abnormalities in prodromal stages. Advances in mitochondrial imaging, high-resolution spectroscopy, extracellular vesicle profiling, and phospho-Rab assays offer promising avenues, but none yet meet the sensitivity or specificity required for routine clinical use.

However, repeated negative neuroprotection trials indicate that mitochondrial involvement in PD pathogenesis does not by itself validate any particular mitochondrial intervention. Successful translation will require demonstrable central target engagement, biologically enriched cohorts, intervention before extensive neuronal loss, and outcome measures capable of detecting restoration of mitochondrial function.

**FIGURE 7:**
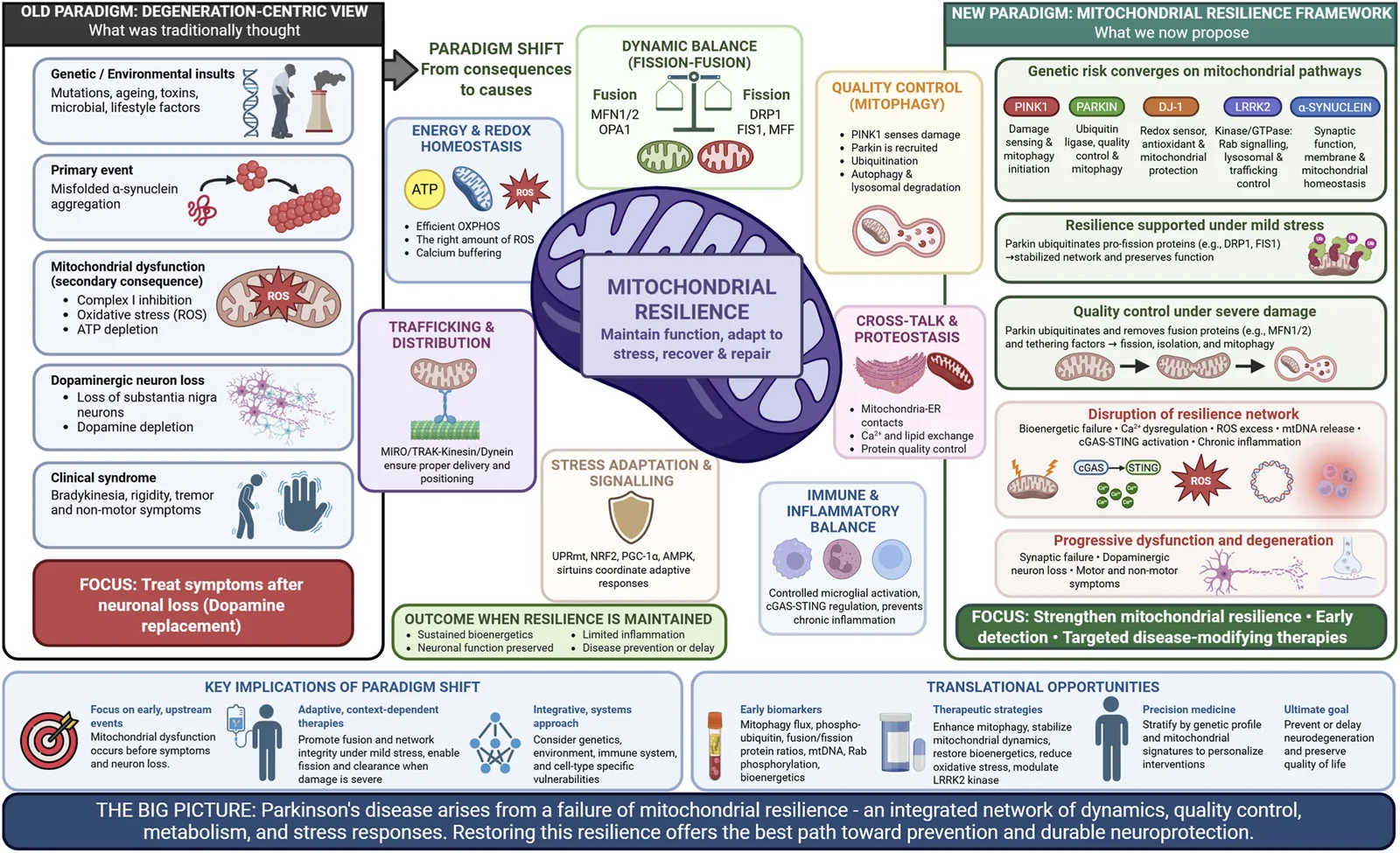
Paradigm shift in Parkinson’s disease: mitochondrial resilience as an integrated systems-level framework. This schematic contrasts a traditional degeneration-centered model of Parkinson’s disease with a mitochondrial resilience framework. In the classical view, genetic and environmental insults are followed by α-synuclein aggregation, secondary mitochondrial dysfunction, dopaminergic neuron loss, and symptomatic treatment after neurodegeneration has occurred. The proposed framework places mitochondrial resilience upstream as a dynamic network that maintains neuronal function through coordinated bioenergetics, redox balance, calcium buffering, fusion–fission dynamics, mitophagy, trafficking, proteostasis, stress adaptation, and immune regulation. Parkinson’s disease–associated proteins, including PINK1, Parkin, DJ-1, LRRK2, and α-synuclein, converge on these mitochondrial and lysosomal pathways. Operationally, mitochondrial resilience refers to the capacity of this network to limit functional disruption during a defined stress, recover bioenergetic and membrane-potential homeostasis after stress removal, and resist transition to irreversible failure. Under mild stress, adaptive quality-control responses preserve mitochondrial function; under severe or chronic stress, disruption of this resilience network promotes bioenergetic failure, calcium dysregulation, reactive oxygen species imbalance, mitochondrial DNA release, innate immune activation, synaptic failure, and dopaminergic neurodegeneration. This systems-level perspective supports a shift toward early biomarkers, patient stratification, and disease-modifying strategies aimed at strengthening mitochondrial resilience before irreversible neuronal loss. AMPK, adenosine monophosphate–activated protein kinase; ATP, adenosine triphosphate; cGAS, cyclic GMP–AMP synthase; DJ-1, Parkinsonism-associated deglycase; DRP1, dynamin-related protein 1; ER, endoplasmic reticulum; FIS1, mitochondrial fission 1 protein; LRRK2, leucine-rich repeat kinase 2; MFF, mitochondrial fission factor; MFN1/2, mitofusin 1/2; mtDNA, mitochondrial DNA; NRF2, nuclear factor erythroid 2–related factor 2; OPA1, optic atrophy protein 1; OXPHOS, oxidative phosphorylation; PD, Parkinson’s disease; PGC-1α, peroxisome proliferator-activated receptor gamma coactivator 1-alpha; PINK1, PTEN-induced kinase 1; PRKN/Parkin, Parkin RBR E3 ubiquitin protein ligase; ROS, reactive oxygen species; STING, stimulator of interferon genes; UPRmt, mitochondrial unfolded protein response.

Ultimately, the goal is a paradigm shift toward mechanism-based prevention and neuroprotection (Figure 7). Pharmacological enhancement of mitophagy, restoration of complex I function, stabilization of mitochondrial dynamics, and modulation of LRRK2 kinase activity are compelling strategies that must be tested in biomarker-stratified clinical trials. Achieving this will require unprecedented integration across molecular neuroscience, computational modeling, clinical neurology, and population genetics.

As the field enters this translational era, the greatest opportunity lies in harnessing mitochondrial biology not merely to explain neuronal death but to interrupt it. The next decade will determine whether early detection and targeted intervention can alter the natural history of PD, transforming a relentlessly progressive disorder into one that is modifiable, predictable, and ultimately preventable.

## Glossary

A30P: alanine-to-proline substitution at position 30 in α-synuclein
Aβ: amyloid-β
ABBV-1088: experimental PINK1 activator
AMBRA1: autophagy and beclin one regulator one
APC: adenomatous polyposis coli
ArfGAP1: ADP-ribosylation factor GTPase-activating protein one
ATF4: activating transcription factor 4
ATP: adenosine triphosphate
BL-918: experimental PINK1/Parkin pathway activator
CaV1.3: L-type voltage-gated calcium channel subtype 1.3
CCCP: carbonyl cyanide m-chlorophenyl hydrazone
CD36: cluster of differentiation 36
cGAS: cyclic GMP–AMP synthase
CNS: central nervous system
CoQ10: coenzyme Q10
COR: C-terminal of ROC
Cys106: cysteine 106
DAMPs: damage-associated molecular patterns
DJ-1: protein encoded by PARK7
DRP1: dynamin-related protein one
E2: ubiquitin-conjugating enzyme E2
ER: endoplasmic reticulum
FIS1: mitochondrial fission one protein
FRA6E: common fragile site FRA6E
G2019S: glycine-to-serine substitution at position 2019
GBA1: glucocerebrosidase one gene
GTP: guanosine triphosphate
GTPase: guanosine triphosphatase
HDAC6: histone deacetylase six
HECT: homologous to E6-AP carboxyl terminus
Hsp31: heat shock protein 31
HtrA2/Omi: high-temperature requirement protein A2/Omi
IBR: in-between-RING domain
iPSC: induced pluripotent stem cell
LManVI: lysosomal alpha-mannosidase VI
LRRK2: leucine-rich repeat kinase two
MARK2: microtubule affinity-regulating kinase two
MFN: mitofusin
MIRO: mitochondrial Rho GTPase
MPP+: 1-methyl-4-phenylpyridinium
MPTP: 1-methyl-4-phenyl-1,2,3,6-tetrahydropyridine
mtDAMPs: mitochondrial damage-associated molecular patterns
mtDNA: mitochondrial DNA
MTK458: experimental PINK1 activator
MTX325: experimental USP30 inhibitor
NADH: reduced nicotinamide adenine dinucleotide
NAC: non-amyloid-β component region
NDP52: nuclear dot protein 52
NFAT: nuclear factor of activated T cells
OPA1: optic atrophy protein one
OPTN: optineurin
OXPHOS: oxidative phosphorylation
OXPHOS-PRS: oxidative phosphorylation polygenic risk score
PARIS: Parkin-interacting substrate
PARK7: gene encoding DJ-1
PARL: presenilin-associated rhomboid-like protein
PD: Parkinson’s disease
PGC-1α: peroxisome proliferator-activated receptor gamma coactivator 1-alpha
PINK1: PTEN-induced kinase one
POLG: mitochondrial DNA polymerase gamma gene
PRKN: Parkin RBR E3 ubiquitin protein ligase gene
Rab: Ras-related protein Rab
RBR: RING-between-RING
RING: really interesting new gene domain
ROC: Ras of complex proteins
ROCO: ROC–COR domain-containing protein family
ROS: reactive oxygen species
Ser65: serine 65
SNARE: soluble N-ethylmaleimide-sensitive factor attachment protein receptor
SNc: substantia nigra pars compacta
SNCA: gene encoding α-synuclein
SQSTM1: sequestosome one
STING: stimulator of interferon genes
TAX1BP1: Tax1-binding protein one
TBK1: TANK-binding kinase one
TFAM: mitochondrial transcription factor A
TIM23: translocase of the inner mitochondrial membrane 23 complex
TOM: translocase of the outer mitochondrial membrane
TRAP1: tumor necrosis factor receptor-associated protein one
TRAK: trafficking kinesin-binding protein
UBL: ubiquitin-like domain
USP30: ubiquitin-specific peptidase 30
VDAC1: voltage-dependent anion channel one
VMAT2: vesicular monoamine transporter two
WD40: WD40-repeat domain
4-HNE: 4-hydroxynonenal
Δψm: mitochondrial membrane potential.
